# CONTROL-CORE: A Framework for Simulation and Design of Closed-Loop Peripheral Neuromodulation Control Systems

**DOI:** 10.1109/access.2022.3161471

**Published:** 2022-03-22

**Authors:** PRADEEBAN KATHIRAVELU, MARK ARNOLD, JAKE FLEISCHER, YUYU YAO, SHUBHAM AWASTHI, AVIRAL KUMAR GOEL, ANDREW BRANEN, PARISA SARIKHANI, GAUTAM KUMAR, MAYURESH V. KOTHARE, BABAK MAHMOUDI

**Affiliations:** 1 Department of Biomedical Informatics, Emory University, Atlanta, GA 30322, USA; 2 Department of Chemical and Biomolecular Engineering, Lehigh University, Bethlehem, PA 18015, USA; 3 School of Information Technology and Engineering, Vellore Institute of Technology, Vellore, Tamil Nadu 632014, India; 4 Department of Computer Science and Information Systems, Birla Institute of Technology and Science, Pilani, K. K. Birla Goa Campus, Sancoale, Goa 403726, India; 5 Department of Chemical and Materials Engineering, San José State University, San Jose, CA 95192, USA; 6 Department of Biomedical Engineering, Georgia Institute of Technology, Atlanta, GA 30332, USA

**Keywords:** Closed-loop simulations, neuromodulation control systems, workflows

## Abstract

Closed-loop Vagus Nerve Stimulation (VNS) based on physiological feedback signals is a promising approach to regulate organ functions and develop therapeutic devices. Designing closed-loop neurostimulation systems requires simulation environments and computing infrastructures that support i) modeling the physiological responses of organs under neuromodulation, also known as physiological models, and ii) the interaction between the physiological models and the neuromodulation control algorithms. However, existing simulation platforms do not support closed-loop VNS control systems modeling without extensive rewriting of computer code and manual deployment and configuration of programs. The CONTROL-CORE project aims to develop a flexible software platform for designing and implementing closed-loop VNS systems. This paper proposes the software architecture and the elements of the CONTROL-CORE platform that allow the interaction between a controller and a physiological model in feedback. CONTROL-CORE facilitates modular simulation and deployment of closed-loop peripheral neuromodulation control systems, spanning multiple organizations securely and concurrently. CONTROL-CORE allows simulations to run on different operating systems, be developed in various programming languages (such as Matlab, Python, C++, and Verilog), and be run locally, in containers, and in a distributed fashion. The CONTROL-CORE platform allows users to create tools and testbenches to facilitate sophisticated simulation experiments. We tested the CONTROL-CORE platform in the context of closed-loop control of cardiac physiological models, including pulsatile and nonpulsatile rat models. These were tested using various controllers such as Model Predictive Control and Long-Short-Term Memory based controllers. Our wide range of use cases and evaluations show the performance, flexibility, and usability of the CONTROL-CORE platform.

## I. INTRODUCTION

Modulating the peripheral autonomic nervous system is a promising approach to regulate the physiological functions of internal organ systems [1]. Closed-loop feedback-control approaches allow adaptive delivery of stimulation to achieve desired outcomes. Designing and prototyping closed-loop neurostimulation systems require flexible and modular simulation environments for experimenting with several components, including i) computational models that represent the organ physiology in response to neurostimulation, also known as the physiological models (PMs), ii) the neuromodulation control systems, and iii) the interaction between the PMs and the control systems. However, existing control systems frameworks and simulation platforms do not facilitate closed-loop Vagus Nerve Stimulation (VNS) control systems modeling without considerable rewriting of computer code, complex installations, and manual configurations.

### A. SPARC PROGRAM

*Stimulating Peripheral Activity to Relieve Conditions* (SPARC) [2] is an NIH-funded research program to develop therapeutic devices for peripheral neuromodulation effectively. SPARC consists of the core components, K-CORE, DAT-CORE, MAP-CORE, and SIM-CORE, to enable the development of peripheral neuromodulation devices. Table 1 summarizes the original components of the SPARC ecosystem.

SPARC has implemented SIM-CORE primarily with the *Open Online Simulations for Stimulating Peripheral Activity to Relieve Conditions* (O^2^S^2^PARC or OSPARC) framework [2], which has been developed by IT’IS.^1^ OSPARC is a web-based platform that allows sharing of SIM-CORE simulations without expecting casual users to install the software toolchain required for each simulation on their local machine. Effective neuromodulation of a complete organ system is a complex task that is difficult to achieve using the existing components of OSPARC.

### B. MOTIVATION

The state-of-the-art, including SIM-CORE/OSPARC or the existing overall SPARC ecosystem, do not have the capabilities to facilitate effective modeling of closed-loop peripheral neuromodulation control systems. CONTROL-CORE, a multi-university^2^ project funded by the National Institutes of Health (NIH) as part of the SPARC Program aims to bridge this gap in the SPARC ecosystem and the state-of-the-art. We develop the CONTROL-CORE framework to become the fifth component of the SPARC ecosystem and bring in the capabilities of seamless and distributed closed-loop simulations for peripheral neuromodulation control systems. By facilitating the modeling of distributed closed-loop neuromodulation control systems, CONTROL-CORE completes the SPARC ecosystem and fills the gap in enabling the modeling of closed-loop VNS control systems.

### C. CONTRIBUTIONS

This paper proposes a protocol and framework to facilitate closed-loop simulations for designing and prototyping peripheral neuromodulation control systems with minimal computational overhead. The CONTROL-CORE protocol, known as concore,^3^ allows modular simulation of controller and PM nodes to run on different operating systems and computing platforms, including OSPARC, local (Windows, macOS, or Ubuntu), Docker, and distributed implementations. The local implementations allow developers to use concore to create simulations from source code with appropriate tools installed locally (e.g., Matlab license). The Docker [3] implementation enables nodes to be containerized [4] so that researchers from diverse backgrounds, such as neurophysiology [5] and radiology [6], may reuse them without manual installation, configuration, source code, or a Matlab license in their host environment. Since the PM may be computationally intensive and/or only available on specific platforms (such as OSPARC), it may be desirable to test such a controller on a limited-resource machine while running the more computationally intensive PM on a different machine via an internet connection. The distributed implementation of CONTROL-CORE enables such an execution over the Internet. Moreover, the distributed mode will allow interfacing closed-loop control algorithms with experimental and clinical systems regardless of the local computing infrastructures.

### D. ILLUSTRATIVE USE CASES

concore is agnostic to the control-system technique and programming environments. It allows the closed-loop control systems modules to be written in several languages (such as Python, Matlab, C++, and Verilog) so that the application developers can code in their preferred language(s) and seamlessly execute them as CONTROL-CORE workflows. We highlight the usability and features of the CONTROL-CORE platform through several illustrative use cases. We adopted the peripheral neuromodulation control systems that we had developed in various languages to use the concore protocol to demonstrate the performance and versatility of CONTROL-CORE platform for designing closed-loop neuromodulation control systems. We tested concore with cardiac PMs, including pulsatile and nonpulsatile rat models. These were connected to Model Predictive Control (MPC) [7] and Long-Short-Term Memory (LSTM) [8]-based controllers. The time to simulate one cardiac cycle in a local implementation is about 0.1 seconds, and in a distributed implementation, a couple of seconds.

### E. PAPER ORGANIZATION

The rest of the paper elaborates on the capabilities of the CONTROL-CORE platform for building closed-loop peripheral neuromodulation control system simulations. Section II presents the background and related work. Section III elaborates the architecture of the CONTROL-CORE platform, including OSPARC, local, Docker, and distributed implementations. Section IV presents the implementation details of the CONTROL-CORE platform. Section V presents use cases and evaluates the functionality and performance of the CONTROL-CORE framework. Finally, Section VI concludes the paper with a summary of the research and future work.

## II. BACKGROUND AND RELATED WORK

The CONTROL-CORE framework constructs closed-loop workflows from multiple programs rather than developing them as a single monolith program. It assumes that separate programs simulate the controller and the PM–they should not be combined in a single program as is typically done on OSPARC. The concore protocol aims to solve the complexities that arise from such separation of PM and controller into two separate programs: i) the controller will eventually be implemented into a medical device and ii) the PM represents the physiological responses of the organ when stimulated by such a device. This section presents the background on closed-loop workflows, OSPARC, and related work on workflow frameworks.

### A. WORKFLOWS

Workflow frameworks facilitate reproducible science by composing modular workflows from services [9]. Standard workflow languages such as Common Workflow Language (CWL) [10] and Workflow Description Language (WDL) [11] are widely used in research and industry as they express complex executions as a workflow composed of reusable service instances [12]. They support creating workflows from containerized services. They enhance the reusability of the programs compared to developing those as a large monolith application [13]. While services can run as stand-alone programs, a workflow requires interaction among the services, ensuring interoperability between their interfaces and input/outputs. Interoperable Application Programming Interfaces (APIs) such as REST interfaces [14] enable chaining the services to compose larger workflows [15].

**FIGURE 1.**
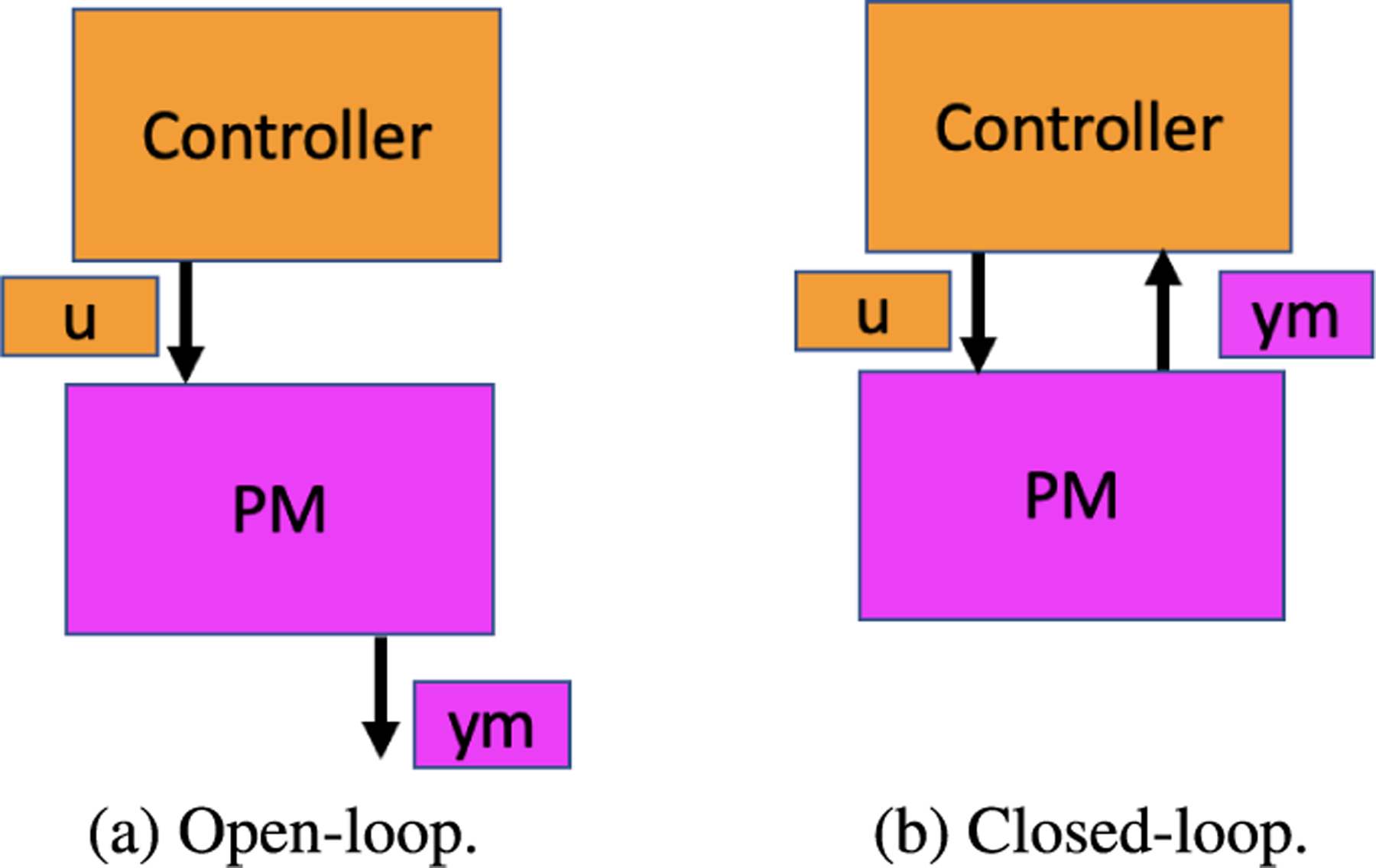
Open-loop vs. Closed-loop.

The workflow standards natively support workflows that have a start and an end step with no directed cycle (dicycle) [16]. These standard workflows can be represented by a Directed Acyclic Graph (DAG) [17], [18]. Such a DAG can denote an open-loop (non-feedback) control, like the one shown by Figure 1a. Here the controller provides stimulation (u) to the PM, and the PM responds with its output (ym). Most of the published models currently on OSPARC either do not have any controller or are open-loop. The input (u) and output (ym) of PMs can be represented as one-dimensional vectors of floating-point numbers. Although internally, PMs may use extensive computations involving large arrays simulating dynamic responses of an organ, the number of sensors and transducers that could ever be implanted in a patient or an animal model is limited. We develop our framework based on this observation that PM communication bandwidth is minimal.

Open-loop control has been shown to be inadequate in various scientific domains such as chemical manufacturing [19] as well as business processes [20], as they consist of feedback loops in the form of dicycles. Figure 1b shows closed-loop control, in which the controller does not issue a predetermined stimulation u, but instead adapts the stimulation based on the feedback it obtains from the PM. Such workflows can be specified by Directed Graphs (DGs) since DGs do not prohibit dicycles, unlike the DAGs. The *interactive services* on OSPARC (such as Jupyter Lab) are not restricted to DAGs and can support feedback loops. However, the *computational services*, such as those vetted by IT’IS staff, are limited to DAGs and therefore also are unsuitable for feedback control.

State-of-the-art workflow frameworks essentially limit their focus to workflows that a DAG can represent. Although research on DG workflows [21] exists, an interoperable generic workflow framework with a high-performance, natively built for the efficiency of closed-loop control systems is still lacking. A closed-loop workflow entails many iterations of each service execution when represented by a series of DAG workflows or even when a DG workflow is natively supported. Hence, an overhead caused by a service execution [22] will quickly add up in executing a closed-loop workflow. Consequently, performance overheads of each iteration of service invocation by a workflow framework [23] will disproportionally impact the performance of closed-loop workflows. Hence, the performance of each service is more crucial in a DG workflow than a DAG workflow to ensure the workflow execution completes in an acceptable time. The overhead and latency caused by the workflow frameworks by executing services multiple times in loops make them unfit for the latency-sensitive peripheral neuromodulation control systems modeling, as closed-loop neuromodulation systems algorithms demand a higher performance with a subsecond latency [24].

Closed-loop peripheral neuromodulation control systems operate at different time scales, depending on the delay of the physiological response. For example, gastrointestinal (GI) closed-loop workflows execute in the orders of minutes, whereas animal heartbeats occur hundreds of times per minute. Although dynamic software-defined workflows [25] can facilitate complex workflows with loops by extending and leveraging the standard workflow languages, their usability and performance are not optimized for peripheral neuromodulation control systems that always consist of loops. Furthermore, such generic frameworks still rely on workflows. Therefore, they are limited by the performance bottlenecks of workflow frameworks. Hardware settings such as ferret-GI [26] feedback require low latency that is not achievable by workflow frameworks at all. This state of affairs significantly hinders the adoption of workflow frameworks in simulating control systems composed of feedback loops.

### B. OSPARC

Although OSPARC provides a User Interface (UI) that allows users to connect the input and output files of simulations with other OSPARC-provided services into “studies” unique to each user, OSPARC was not designed with the concept of feedback control as something that users might wish to experiment with. To publish a simulation on OSPARC, a researcher needs to provide source code to IT’IS, who manually vet the code and containerize it to be compatible with the underlying architecture of OSPARC. This process can take several days. Such containers may only run on the OSPARC platform.

Two of the services that OSPARC provides are Jupyter Lab [27] and Jupyter Notebook [28]. Jupyter Lab allows a user to develop Python and Octave code directly on OSPARC without needing the assistance of IT’IS. Octave [29] is an open-source workalike of the proprietary Matlab [30] language supporting only a subset of its features. Jupyter Notebook, which provided somewhat similar functionality for Python, has been deprecated. Jupyter Notebook or Jupyter Lab would be instantiated with the same graphical UI as other OSPARC-provided services: the user double-clicks to choose Jupyter Lab or Notebook, which appears in the user’s study as a rectangle. The user may rename this rectangle, which acts as a node in a DG. The user can then provide links to other nodes, which are visibly unnamed edges in the DG.

Our goal for CONTROL-CORE is to be a framework for peripheral neuromodulation control systems simulations with feedback control as its central concept. OSPARC allows interactive nodes such as Jupyter Lab to interconnect with each other. IT’IS has recently enhanced the underlying OSPARC engine by revising it to use sidecars to transfer the changed Jupyter Lab output files to the input of other nodes every few seconds. We have observed it taking as long as 10 seconds, although 1–2 seconds is more typical. Therefore, currently, a Jupyter Lab PM node could accept its input edge from a Jupyter Lab controller node (telling the organ what electrostimulation is being provided) while simultaneously the PM’s output (e.g., heart rate) may be input to the controller, thereby closing the loop. By leveraging this recent enhancement to OSPARC, we developed concore, which is described in greater detail in the next section.

## III. SOLUTION ARCHITECTURE

This section describes how the concore protocol allows running modular workflows on different computing and programming environments without having to change the source code for nodes.

### A. THE PROTOCOL

The concore protocol is simple code, which could be written in any language that supports rudimentary file operations. It polls and sleeps to wait until a change occurs before proceeding with computation. The computation within each node is based on both the input data from the file and the internal state maintained locally within that node. The computation result is written out to another file that other nodes will be waiting for. The concore style of coding is like event-driven approaches used in Hardware Description Languages (HDLs) [31], allowing graphs more complicated than a simple controller-PM system to be resolved.

**FIGURE 2.**
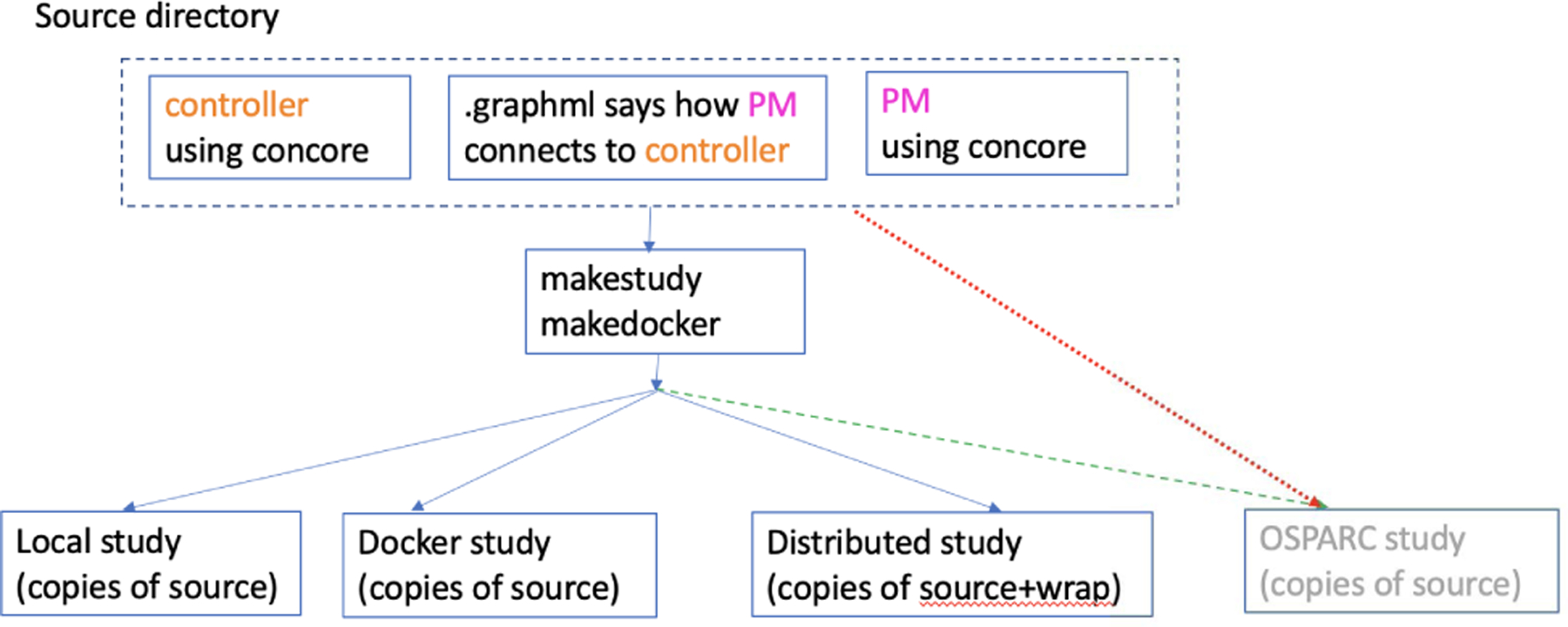
A workflow following the concore protocol.

An application developer can develop a CONTROL-CORE workflow from programs such as PMs and controllers. The user specifies the workflows through a workflow definition (a graphically composed GraphML [32] file) illustrating the communications across the programs. Existing programs can be adapted to follow the concore protocol to compose the workflows. If the programs do not exist, the application developer can write them first, following the concore protocol from scratch. Figure 2 illustrates how a concore workflow is developed, built, and executed across multiple platforms.

There are two possible ways to develop a workflow for OSPARC: the red dotted arrow indicates uploading the source code directly to OSPARC as described in the next section; the green dashed arrow indicates using the same tools on OSPARC as used for the local implementation described later.

We have implemented the concore protocol in Python, Octave, and (for systems outside of OSPARC that possess the appropriate license) Matlab. We also partially support Posix shell scripts to facilitate the running (on systems without the license) of previously-compiled Matlab, using the Matlab Compiler Runtime (MCR) [30]. In addition, we support languages not supported on OSPARC, including Verilog (to model the hardware limitations of limited-resource controllers) and C++.

### B. OSPARC EXECUTION

In the typical case (like Figure 1b) we might have a controller whose input from the PM is a file called “ym”. Using the concore protocol, this takes only two lines of Python:

**while** concore . unchanged ( ) :
ym = concore . read ( 1 , “ym” ,initialym)

or similar Matlab/Octave:

**while** concore_unchanged ( )
ym = concore_read ( 1 , “ ym “ , initialym ) ;
end

or C++^4^:

vector<**double** > ym ;
**while** ( concore . unchanged ( ) ) { ym = concore . read ( 1 , “ym” ,initialym ) ; }

where ym is the variable that contains the updated value read from the file “ym”, and the number 1 is a port number. (Since OSPARC allows multiple input ports with multiple files per port, the concore protocol allows multiple concore.read calls inside the concore.unchanged loop.) The concore.read encapsulates file and sleep operations, and keeps track of the previous value(s) so that concore.unchanged will cause the concore.read to re-execute until the new value of ym is different than the previous value. Because the file may not exist at the start of the simulation, an initialym must be provided (eliminating the chicken-or-egg dilemma between the two nodes). The amount of real-time for the sleep call is configurable with concore.delay.

The data file transferred in the concore protocol is required to be the textual representation of a one-dimensional list (array) of floating-point values, with a simulation time stamp appended on the left. In other words, “[“followed by the simulation time and then a comma-separated list of numbers terminated by “]”. This representation is native to Python and directly acceptable to Matlab. We developed a parser of this format for other languages. The concore.read automatically strips the simulated time from the input data and uses this to determine concore.simtime. (In the case of multiple inputs, this will be the maximum of all possible times). The remaining one-dimensional vector will be returned to the user. If a control system formulation requires two-dimensional data (e.g., column vectors), it is the user’s responsibility to convert this data to the format as above. The initialym value is the full-textual representation that would have been found in the file if it had existed (a string including the “[“, simulation time and “]”).

**FIGURE 3.**
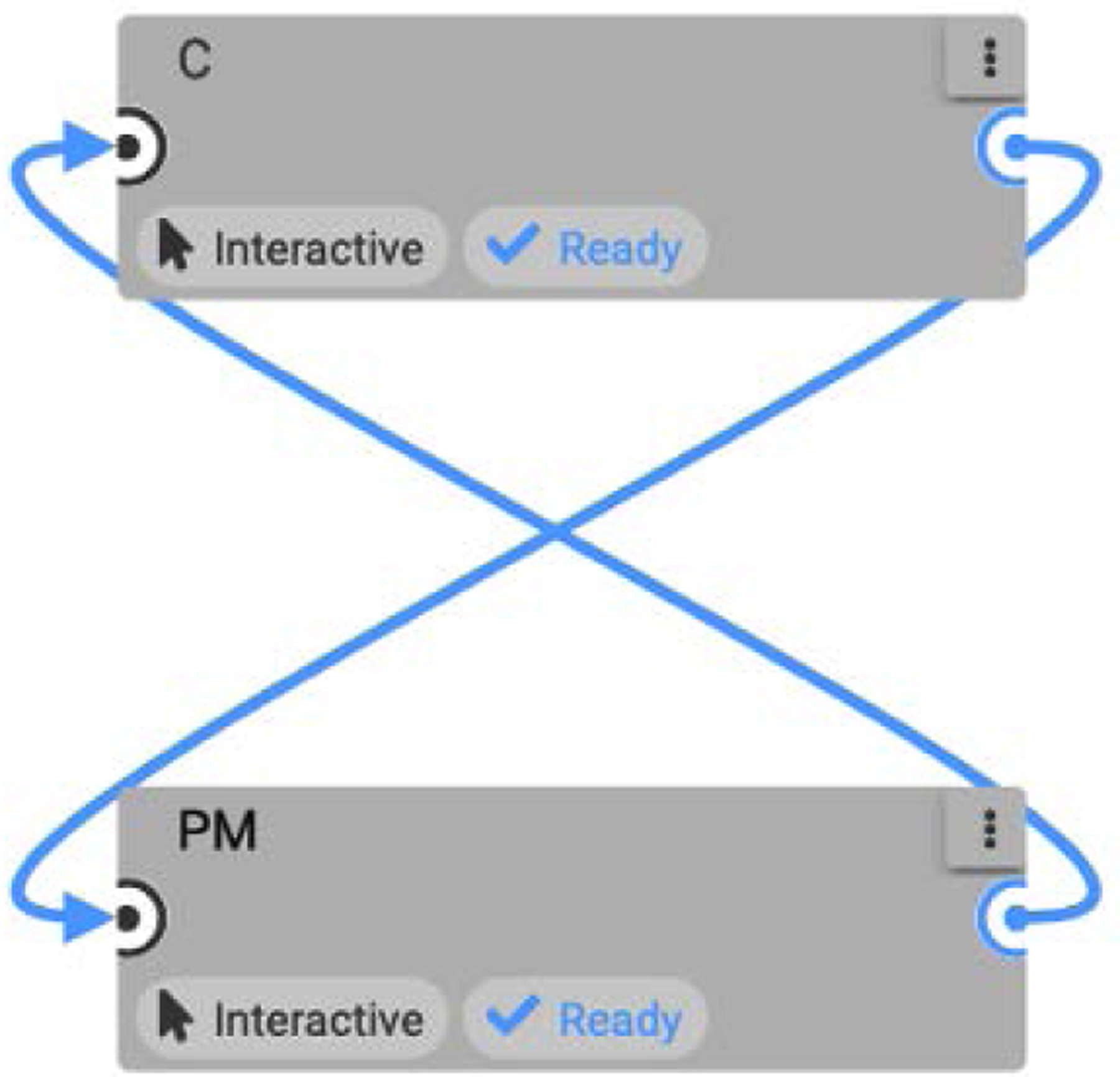
OSPARC study with C and PM.

Associated with the same file “ym” in the other node (the PM) is

concore . write ( 1 , “ym” , ym , delta )

which again encapsulates the file operations required. In the OSPARC implementation, this file actually resides in a directory /outputs/output_1 that IT’IS uses for sidecar file transfer. The connection between the two concurrently running Jupyter Lab nodes is made by the IT’IS sidecar file-sharing mechanism denoted by the edge from PM to Controller (C) shown in Figure 3.^5^ This causes “ym” to be transferred to /inputs/input_1 in the other Jupyter Lab node (C), which is where the concore.read shown above will look for this file. The analogous approach that concore uses on non-OSPARC implementations will be described later.

The variable ym could be replaced with any expression that returns the correct sized vector. The concore.simtime is automatically concatenated to the string written to the file. The parameter, delta, allows the advancement of simulation time. Advancing the simulation time with non-zero delta in at least one node in the loop is necessary to trigger other nodes to continue the simulation. Part of the reason simulation time is included in every file is that the actual vector (excluding the time) may remain identical during consecutive times. Without explicitly including the time in the file input to a node, the node could not know it is supposed to be triggered.

Similar concore calls are associated with the other edge in Figure 3, which transfer a file “u”. In the PM:

**while** concore . unchanged ( ) :
u = concore . read ( 1 , “ u “ ,initialu)

and in the controller:

concore . write ( 1 , “ u “ , \ ldots )


Because of the concurrency of nodes, a potential for race conditions exists, where the concore.write in the one node is not finished at the time concore.read in the other node is accessing the same file. Recall that concore.read polls for a change in the file (in the most recent example, “u”). At startup, this file may not exist, and if there is a file exception, our code assumes the default value (initialu) to start the simulation. Later, concore assumes that *only* one of three things happens: 1) the file is identical to the last time it was read (and so the polling continues); 2) the file’s contents have changed (and so the polling stops); or 3) the file has zero bytes because the file has been reopened for write but has not yet been overwritten (and again the polling continues). If this assumption is satisfied, concore synchronizes the nodes correctly. This assumption is valid when we run the two nodes on non-OSPARC implementations described in the next section, but we occasionally noticed a brief moment when the OSPARC sidecar file sharing removed the old version of the file from a node before copying the new file from the other node. This caused the default value to be inserted in the middle of a simulation, destroying the validity of the result. The staff at IT’IS corrected this bug so that concore operates flawlessly on OSPARC.

### C. LOCAL EXECUTION

The CONTROL-CORE project began with the goal of finding a way to use feedback control within OSPARC; however, the scope of CONTROL-CORE is larger than a programming technique to be used only on OSPARC. We designed concore so that the same code that operates on OSPARC will work when it runs on other platforms, such as MS Windows, macOS, Ubuntu, or Docker.

**FIGURE 4.**
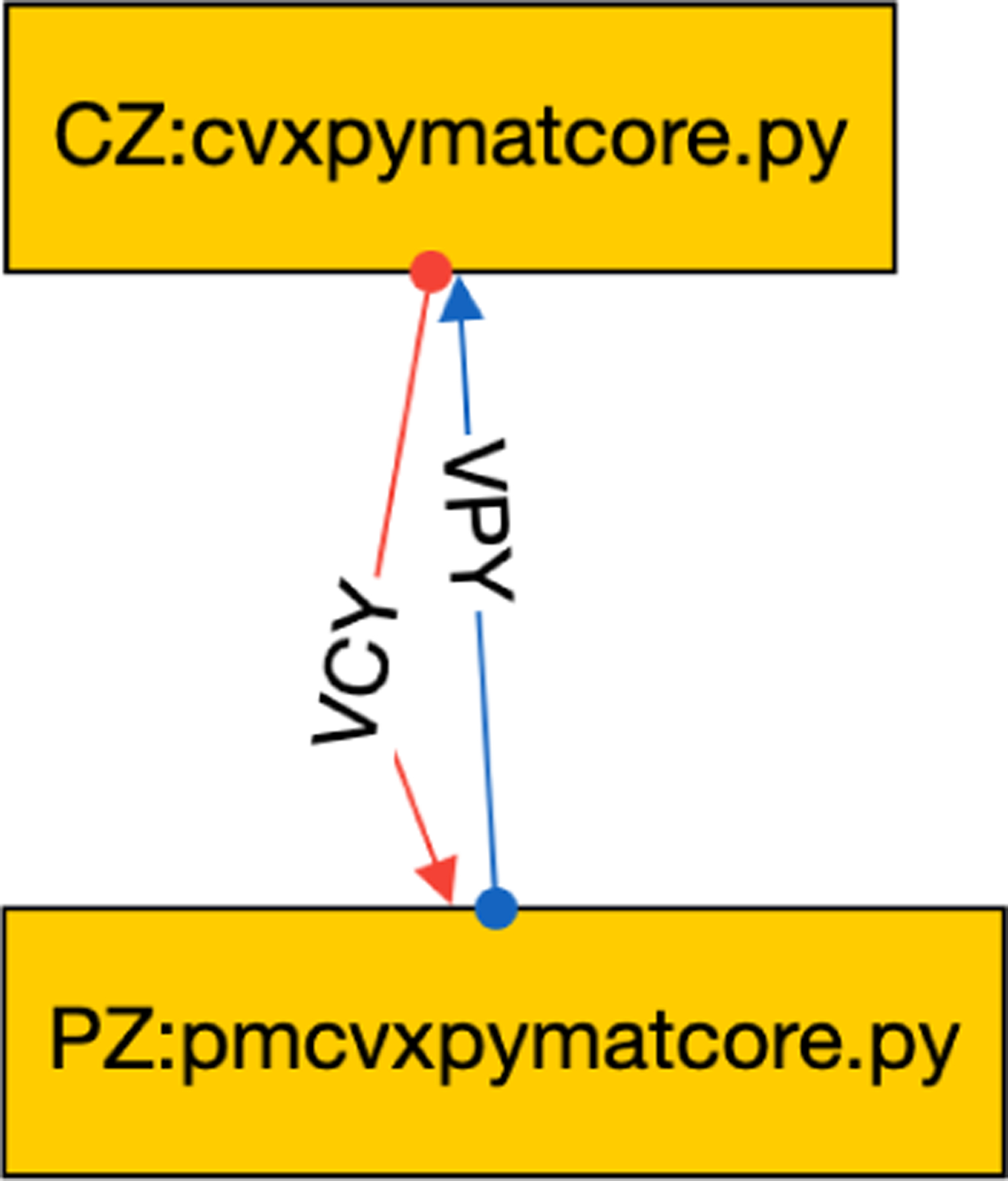
A concore workflow.

To make the framework user-friendly, we needed to replace the OSPARC UI and its associated runtime engine for the local execution on non-OSPARC environments. We considered using graphical front ends provided for standard workflow languages for the UI. However, the inherent feature of all such workflow languages is that they are based on DAGs, which is incompatible with closed-loop control. We built a minimal workflow composer that allows users to graphically create more diverse workflows, such as the one shown in Figure 4, and store them in a GraphML format. The nodes in Figure 4 show the instance names of the controller (CZ) and physiological model (PZ) as well as the associated Python programs. The edges that connect the nodes also have unique labels.

The basic feature concore relies on is some form of file sharing between nodes. When controller and PM run as independent processes on a local Posix operating system (either macOS or a version of Linux such as Ubuntu), the file-sharing can be accomplished by using a symbolic link (ln -s) between the input and output directories. We can simplify the paths a bit from what OSPARC uses, making the input port 1 directory for the controller (let’s call it CZ this time) be ./CZ/in1. This is symbolically linked to a directory that is also symbolically linked to the output port 1 directory for the physiological model (let’s call it PZ this time), which is ./PZ/out1. Similar symbolic links occurs for ./PZ/in1 and ./CZ/out1. The current directory (.) in this example is the directory on the local Posix machine equivalent to an OSPARC study. Processes running Windows support a similar concept but use “\” in the paths being symbolically linked (by mklink). The visual representation of the GraphML allows us to hide these implementation details from the user.

The GraphML^6^ (Figure 4) is processed by our command-line tool called makestudy that the user invokes as

. / makestudy sdir / example . graphml

where sdir is a directory that contains the workflow (example.graphml here) along with the source files it references. This creates a directory example with the same name as the workflow that has several scripts. In addition to the scripts, makestudy creates a ./src directory that contains a copy of the relevant source files, the Python (concore.py), C++ (concore.hpp), Verilog (concore.v) or Matlab (concore*.m and import_concore.m) library, and additional support files. The most important script that makestudy generates is a script (known as ./build) that sets up the necessary symbolically-linked directories. (See Appendix for details.) Our tool also generates another script (known as ./run) that initiates running the two programs (./CZ/cvxpymatcore.py and ./PZ/pmcvxpymatcore.py) in separate processes (analogously to what happens in an OSPARC study). Here is the ./run script generated by makestudy using Figure 4 as the GraphML file for Posix:

( **cd** CZ ; python3 cpymat . py >concoreout . txt& **echo** $! >concorepid )&
( **cd** PZ ; python3 pmpymat . py >concoreout . txt& **echo** $! >concorepid )&


In each node (in this example, CZ and PZ), this script creates concorepid (the process id of the node used by ./stop) and concoreout.txt (the output of the node). There is not an easy equivalent to $! in Windows to find the process id of the node to used by stop.bat. Instead, at runtime concore.py or its Matlab equivalent (import_concore.m) create batch files that do taskkill /F /PID of the process id for the currently running process if that process detects it is running under Windows. Again, these Windows batch files will be created for each node, and stop.bat invokes these scripts.

The tool also generates: a script named ./maxtime which allows the user to specify the maximum simulation time in concore.maxtime for each edge directory (e.g., ./VCY and ./VPY of Figure 4); a script named ./debug which allows the user to interactively debug each node in separate windows; a script named ./stop which stops the nodes from running if they do not terminate; and a script named ./clear which erases the files used for sharing between nodes so that the simulation can be restarted.

As shown in Figure 4, the GraphML file gives more information than was shown in the OSPARC UI. Each node is labeled with a name (corresponding to a directory that will contain that node). This is typically followed by a colon and the source file’s name that will run in that node. (On OSPARC, the user must have manually uploaded this file to Jupyter Lab prior to running it). Our tool notes the extension of the source file (Python, Octave, Matlab, C++, and Verilog have different requirements to run). Also, we require each edge in the GraphML file to be labeled with a unique name. These names (VPY and VCY in the example of Figure 2) correspond to the actual directories that contain data files ym and u, respectively.

**FIGURE 5.**
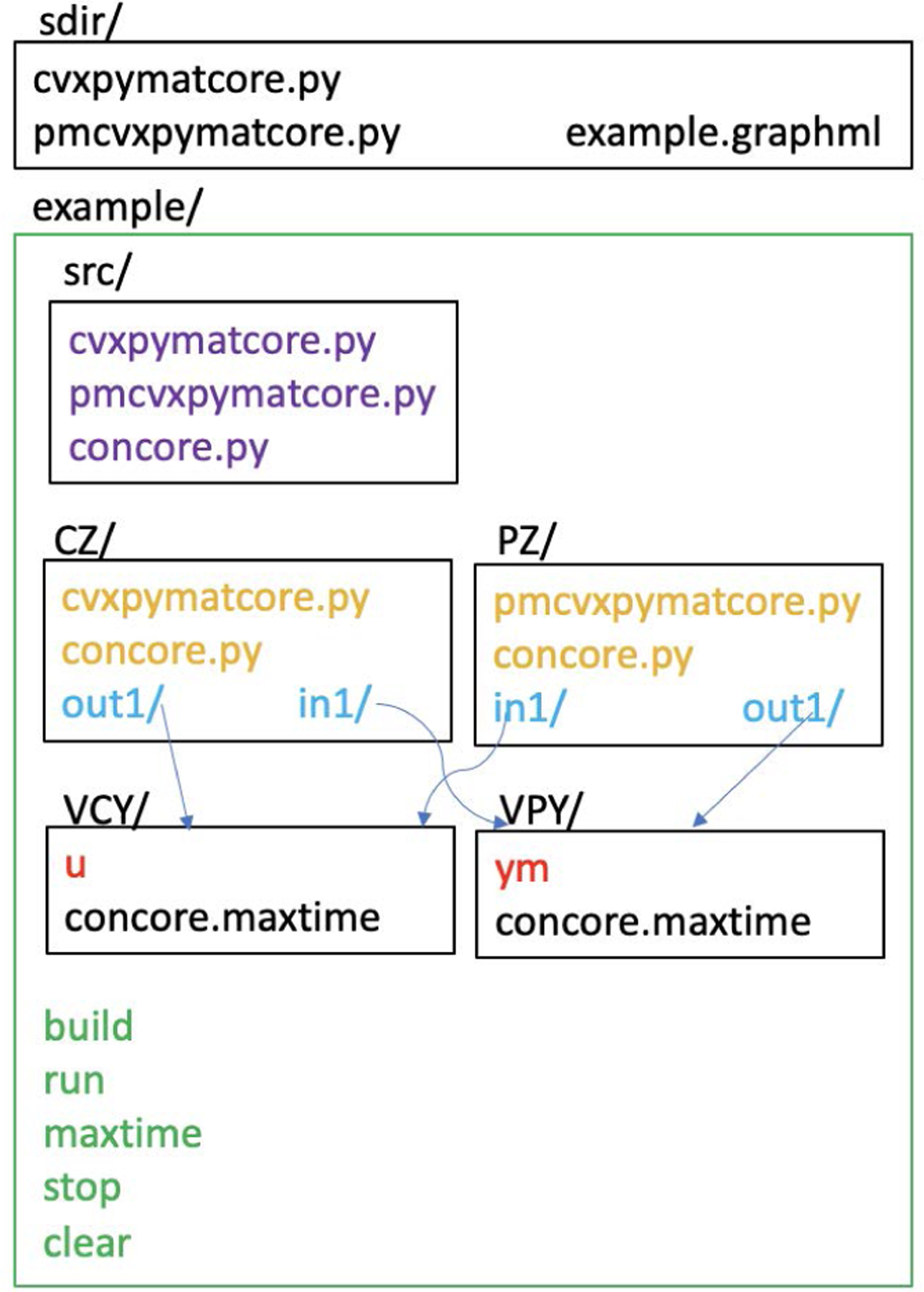
Files and directories of an illustrative local concore execution.

Figure 5 shows the files and directories of a sample local concore execution. example directory has scripts (green) and src subdirectory with source files copied from sdir (purple). After ./build it has subdirectories (CZ, PZ) with another copy of source files (orange) and symbolic links (blue) to subdirectories (VCY,VPY) that transfer data files (red). ./PZ/in1 and ./CZ/out1 are both symbolically linked to ./VCY which contains u. Likewise, ./CZ/in1 and ./PZ/out1 are both symbolically linked to ./VPY which contains ym.

### D. CONTAINERIZED EXECUTION

In addition to local implementation using operating system symbolic links, concore supports a containerized execution with Docker:

. / makedocker sdir / example . graphml


Instead of symbolic links within the same file system, concore accomplishes file sharing for the containerized execution using Docker volumes (VCY and VPY in the illustrative execution presented in Figure 5) and Docker containers (PZ and CZ in Figure 5). In the example directory, makedocker creates the Dockerfiles needed to build the images docker-cvxpymatcore and docker-pmcvxpymatcore. It also creates the ./run needed to run them:

sudo docker run --name=CZ -v VCY : / out1 -v
VPY : / in1 : ro docker–cvxpymatcore\&
sudo docker run --name=PZ -v VPY : / out1 -v
VCY : / in1 : ro docker-pmcvxpymatcore\&


The ./stop and ./clear scripts accomplish a similar effect as their non-Docker counterparts, with the extra restriction that to be successful, ./stop must be done before ./clear. This is because ./stop does docker stop and docker rm. The ./clear, which does docker volume rm, fails if there are containers attached to the volumes associated with GraphML edges, so the ./clear must happen after ./stop.

The ./maxtime script is more involved than its non-Docker counterpart since docker cp only allows copying files to containers, not directly to volumes. It is necessary to place concore.maxtime in volumes because the user invokes the ./maxtime script before the ./run script, and so at that point, the containers do not exist.^7^ To broadcast concore.maxtime to all volumes, the Docker ./maxtime script momentarily builds, runs, and destroys a container called concore that mounts all volumes. Using paths through this container, the user-specified maximum time is copied to each volume just prior to the user invoking ./run.

A different option, which departs from the requirement of providing source code for every node, pulls previously-built Docker containers for nodes when the language extension is not given for a node in the GraphML file. This will be useful for general-purpose controllers that naïve users can instantiate without having to understand their internal operation. In this option, the ./run script is similar to those above, but pulls the image from the Docker hub, and the ./build does not do anything (at least as regards the node pulled from the Docker hub). Obviously, this option is only useful in the Docker implementation.

**FIGURE 6.**
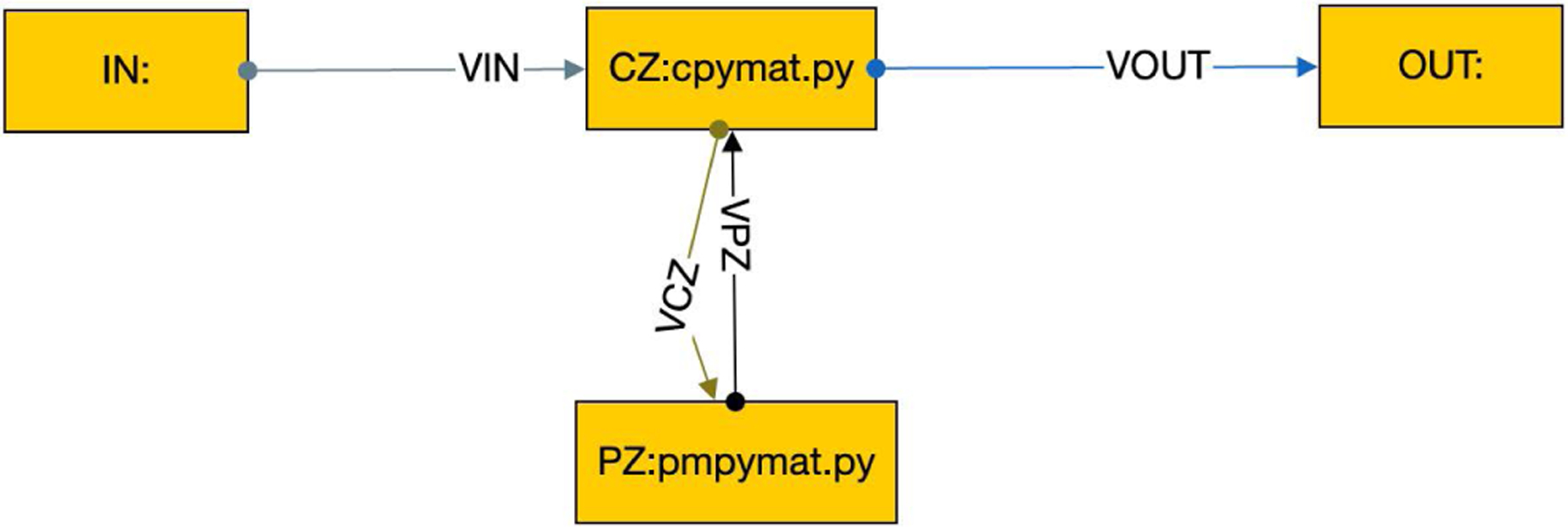
Examples of null nodes.

The user provides source files for every node in the GraphML file in the typical case. Another exception to this requirement is what we call a *null node*, where the node name is followed only by a colon. Since the contents of Docker volumes exist prior to and after the Docker run command, such volumes can provide inputs and outputs to the overall system by connecting one side to a null node. Null nodes do not correspond to containers. Figure 6 illustrates a four-node system of which two are null nodes, IN and OUT. This is useful because the null nodes allow additional Docker volumes, VIN and VOUT as edge labels, but otherwise is similar to the two-container system of Figure 4.

Here is the corresponding ./run script generated by makedocker for the GraphML file of Figure 6:

sudo docker run --name=CZ -v VCZ : / out1 -v
VOUT : / out2 -v VPZ : / in1 : ro -v VIN : / in2 : ro
docker−cpymat\&
sudo docker run --name=PZ -v VPZ : / out1 -v
VCZ : / in1 : ro docker-pmpymat\&


Because CZ has two outputs, concore.oport[‘VCZ’] will be 1 and concore.oport[‘VOUT’] will be 2. Likewise, concore.iport[‘VPZ’] will be 1 and concore.iport[‘VIN’] will be 2.

**FIGURE 7.**
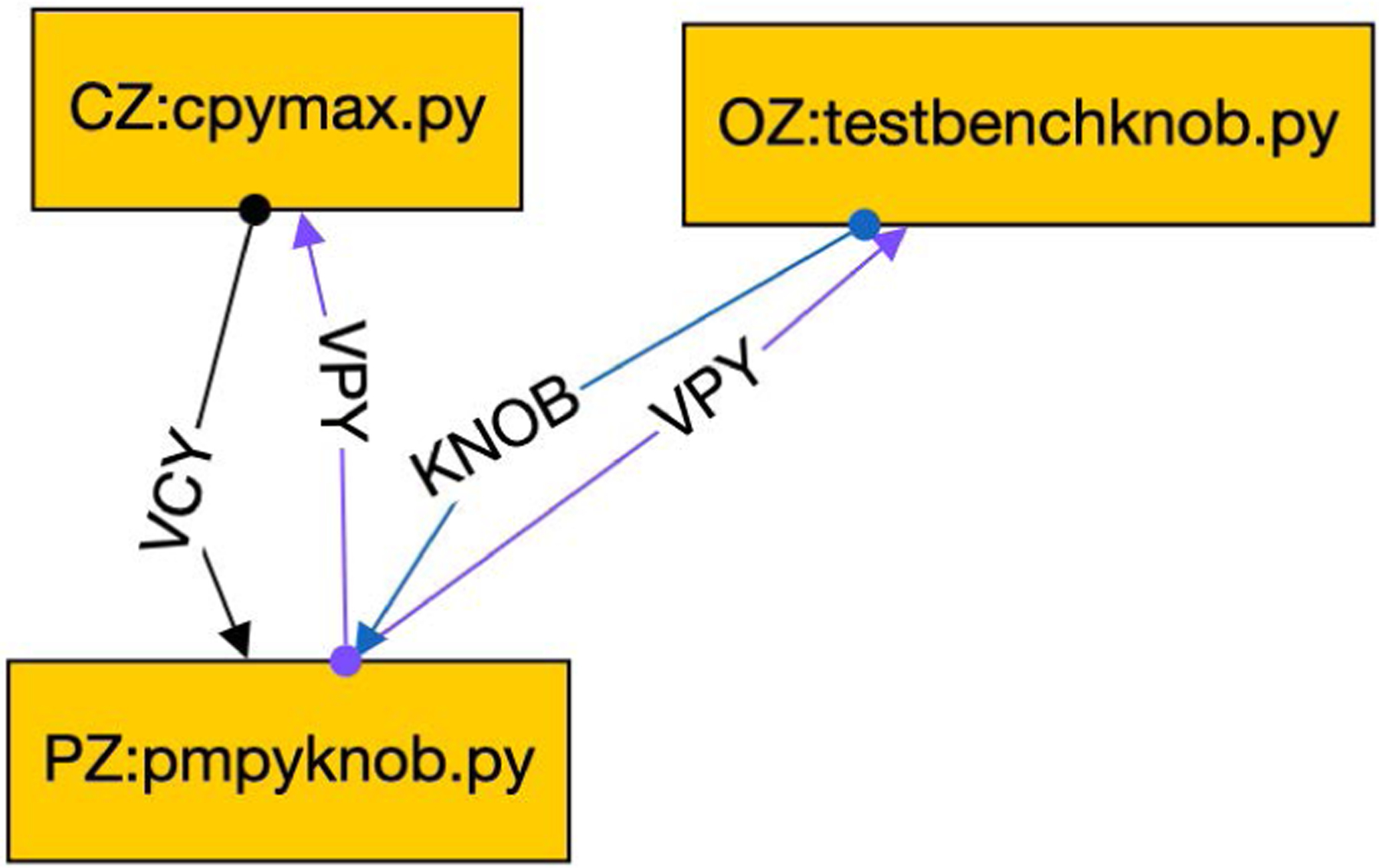
Testbench node modifies knob to PM.

The approach illustrated in Figure 6 is useful when the input file is to remain unchanged during the entire simulation. A more complicated situation occurs when a user wishes to vary some experimental parameters during the simulation, for example, whether the PM exhibits a healthy or diseased behavior. Although such behavioral change could be hard-coded into the PM, doing so would be a poor choice for a PM that will be reused in different contexts. Instead, CONTROL-CORE supports *testbench nodes* that output knob files to allow a user to customize the behavior of the system, as shown in Figure 7. The KNOB edge is an optional input to the PM. This PM is written more generally than before to use a default value if this edge does not exist. The testbench node does not set the simulation time pacing of the system (which is still determined by the feedback loop between CZ and PZ). Rather OZ observes the PM output, and based on conditions selected by the user, changes the KNOB accordingly. This is particularly useful when the CZ and PZ are pre-built Docker images for which the user cannot modify the source code. Instead, the user gives a simple source code for OZ to specify the circumstances in which the KNOB will vary.

### E. DISTRIBUTED EXECUTION

**FIGURE 8.**
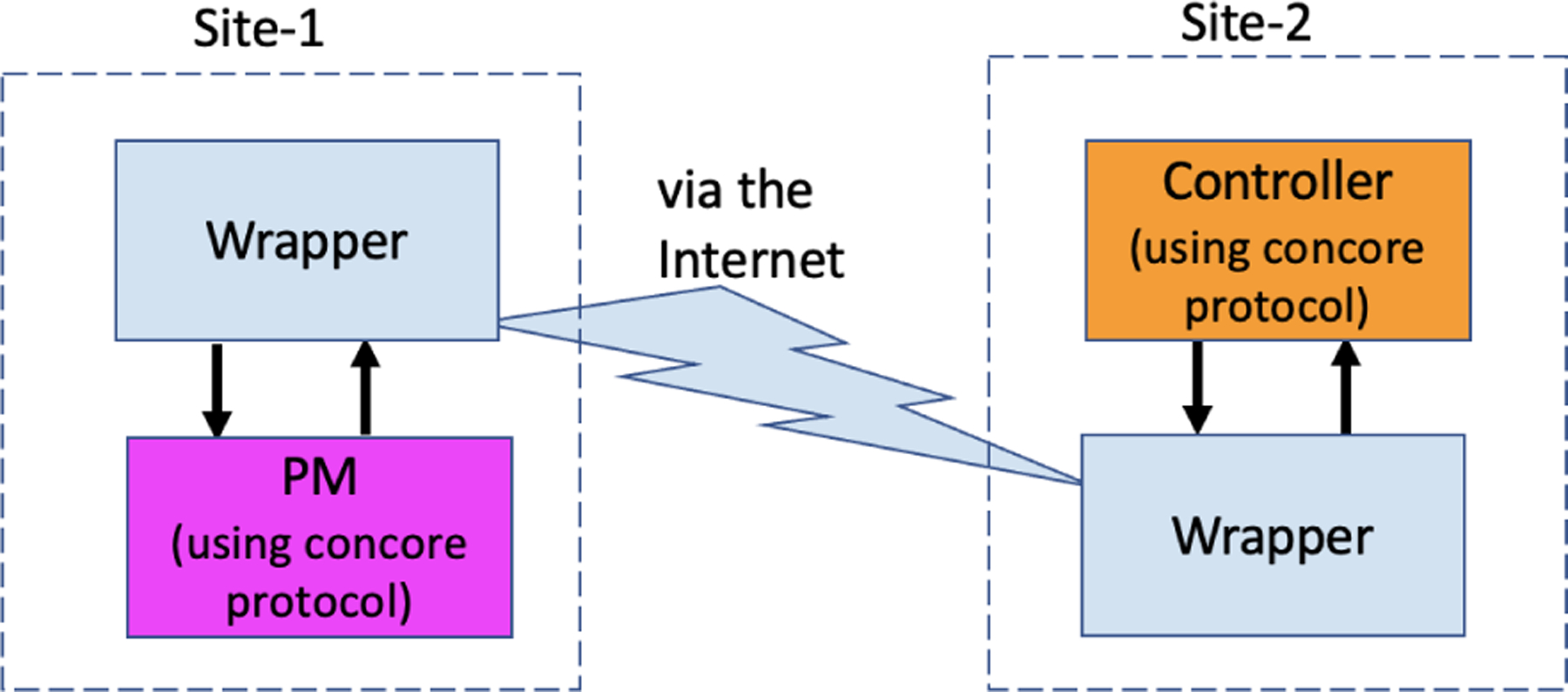
Communication via the Internet.

The concore protocol enables running a controller with the PM together on the same platform, whether that platform is OSPARC, Windows, Ubuntu, macOS, or Docker. But in practice, a successful controller will eventually be implemented in an embedded processor (so that a custom circuit can be implanted in an animal experiment or a patient in the future). Since the PM may be quite computationally intensive and/or may only be available on certain platforms (such as OSPARC), it would be desirable to test such a controller on a limited-resource machine (similar to that which will be implanted) while running the more computationally intensive PM on a different machine via an internet connection. Therefore, the CONTROL-CORE platform supports a distributed execution to facilitate workflows that span multiple servers from various organizations. How can we distribute the PM and controller onto separate sites? In the abstract, it involves a data transfer via the Internet, as illustrated by Figure 8, but with the concore library being used at each site.

The distributed implementation communicates via the Internet with the support of “wrappers” so that a controller can be local while the PM can be remote (e.g., running on OSPARC). We introduced a wrapper in place of the controller and PM on each host, thus seamlessly separating and distributing the execution to two sites. The wrapper in site-1 takes the placeholder position of the controller, whereas the wrapper in site-2 assumes the position of the PM. With this approach, the local and distributed simulations can be made seamless without hindering the user adoption of either approach. The source code for PM and controller does not change whether both run locally at a single site without wrappers or are distributed between two sites using wrappers.

**FIGURE 9.**
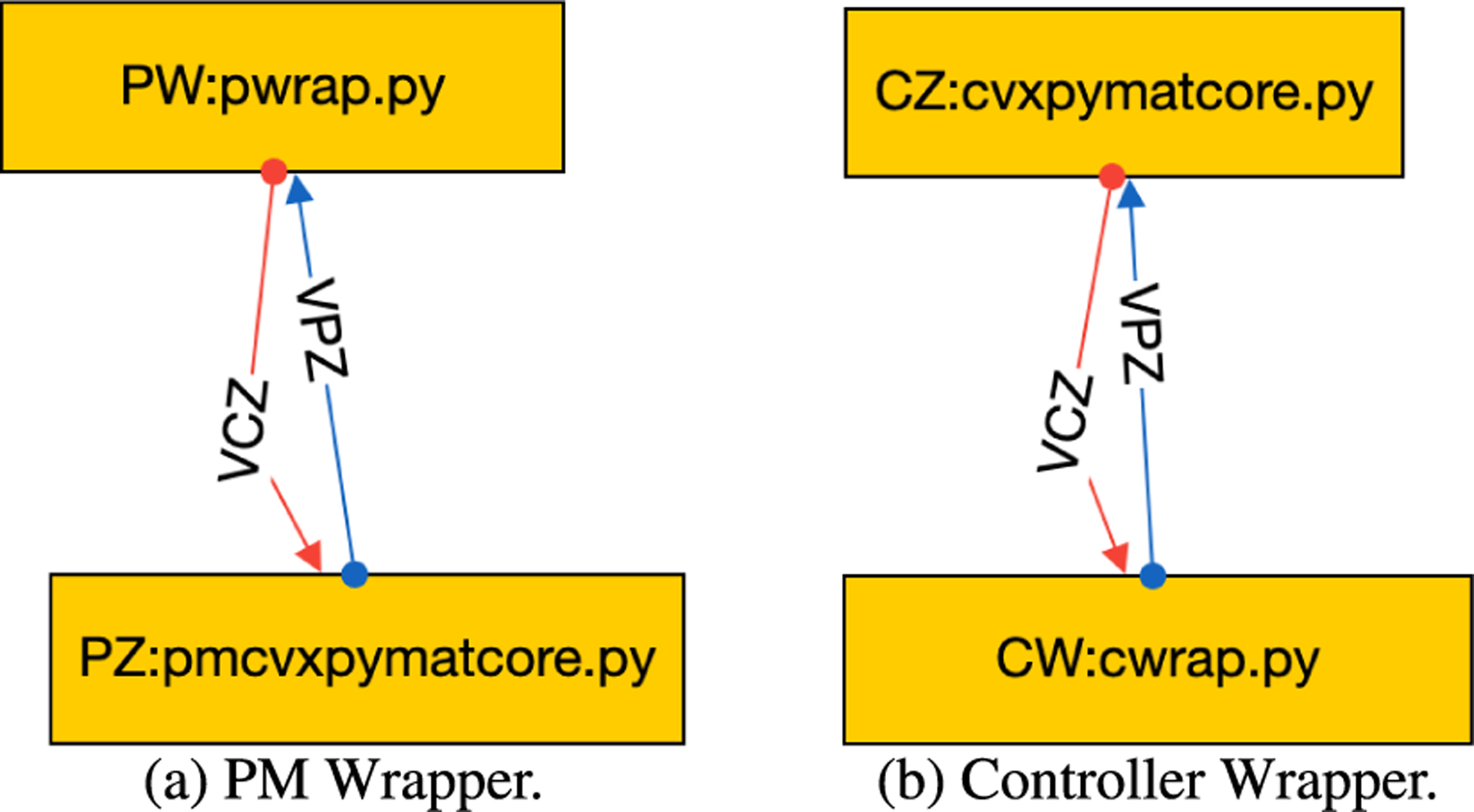
Wrappers.

The GraphML file that has the wrapper for the PM (node name PW, source file pwrap.py) is shown in Figure 9a. The GraphML file that has the wrapper for the controller (node name CW, source file cwrap.py) is shown in Figure 9b. In these figures, the nodes PZ and CZ and associated source files are identical to the earlier example. Instead of using the concore protocol to communicate directly between PZ and CZ, they use the concore protocol to communicate with their associated wrappers.

We considered three alternative approaches to implement the distributed execution of CONTROL-CORE. First, we considered a peer-to-peer architecture with site-1 and site-2 as peers. However, such an architecture appeared overengineered given the limited bandwidth required by CONTROL-CORE. Next, we considered a client-server architecture. Finally, we settled on a Mediator-based architecture.

#### 1) CLIENT-SERVER ARCHITECTURE

The client-server architecture provides a straightforward implementation, using RESTful POST requests for the distributed execution. In this approach, the user runs the wrapper of the controller as a server in a cloud server or a remote server. The user runs the wrapper of PM from the client site, such as OSPARC (by logging onto OSPARC and initiating a study containing the wrapper) or a local machine. The client site uses Python’s requests [33] module. Since the Python request module already exists in OSPARC, OSPARC can run the PM as an ordinary client, not requiring any modification by IT’IS. However, if all the controller computations are performed in the cloud, this approach will be more CPU intensive and costly. Furthermore, this could impose a maintenance challenge should more workflows (than a simple controller) be introduced.

#### 2) MEDIATOR-BASED ARCHITECTURE

**FIGURE 10.**
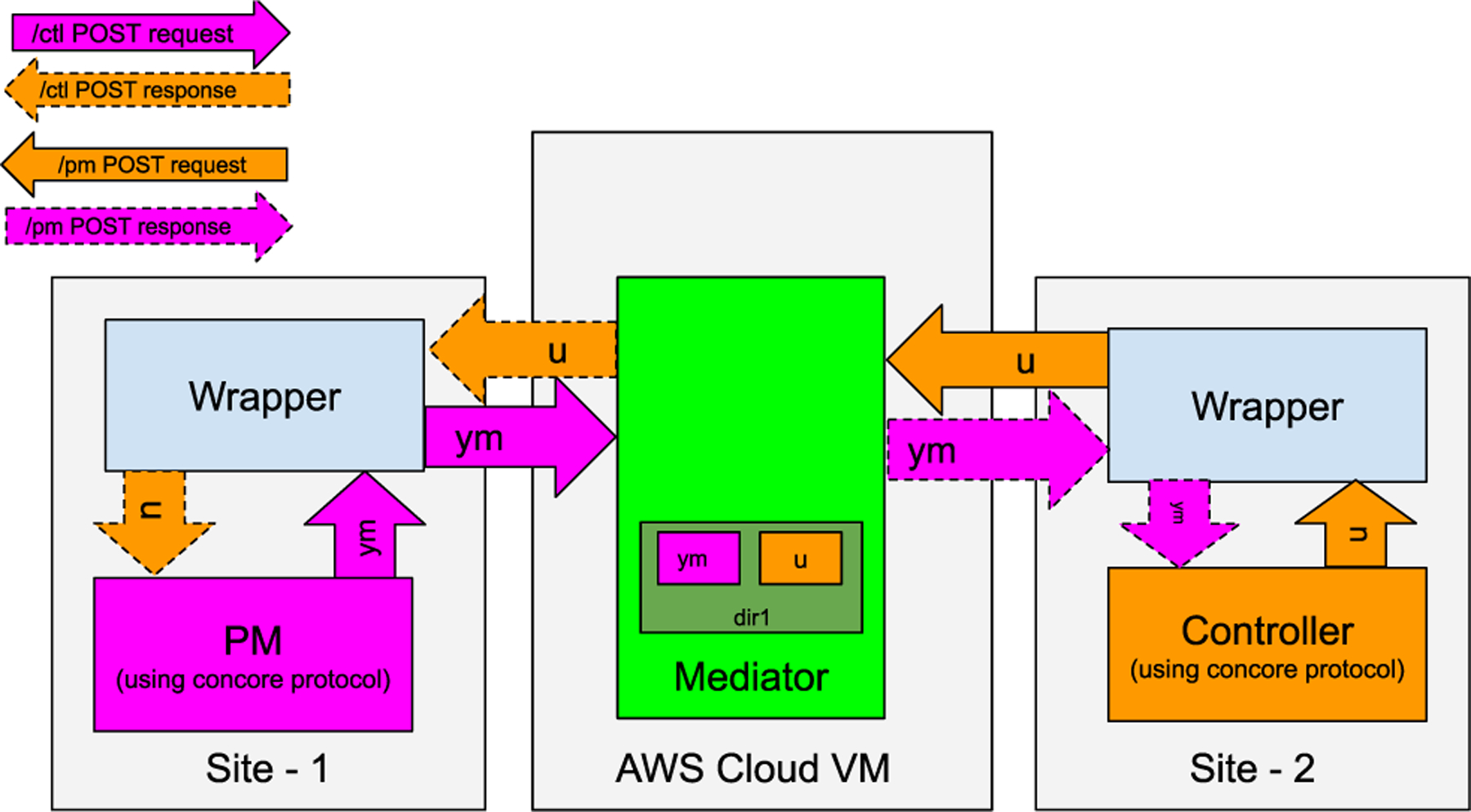
The Mediator-based architecture.

In the chosen Mediator-based architecture, as depicted in Figure 10, a central cloud VM consists of Mediator,^8^ a lightweight RESTful server application. We developed a secure and multitenant implementation of a Mediator [34] to facilitate loosely-coupled communications between the PMs and controllers via the wrappers. The Mediator facilitates the execution of workflows using the respective wrapper routines of the PM and the controller. We hosted the Mediator in an Amazon Web Services (AWS) cloud server to facilitate communications between the distributed PM and controller nodes deployed across various sites.

We developed the Mediator with Flask [35], a lightweight web-service engine that is often used to build web applications from Python classes quickly. The Mediator functions as an intermediary that accepts the files from the POST requests. All services (PM and Controller, in the depicted case) of a workflow acquire their files through POST requests posted to the Mediator by the other services composing the workflow (i.e., in the illustrated case, the PM gets its input files from the controller; the controller gets those from the PM). The Mediator temporarily stores them in a user-specified directory (such as “dir1” in Figure 10) to exchange across the services of the workflow. The Mediator performs no computation except matching the outputs of PM and controller from different sites. Hence, it is cost-effective, less CPU intensive, and more scalable than a client-server model.

The Mediator approach allows all the service nodes to be equal, rather than following an asymmetric client-server model with having to decide on either PM or controller as the server and the other one as the client. Both site-1 and site-2 can be client sites such as OSPARC or a local machine. For example, this allows a user of an OSPARC PM to “jailbreak” and connect it to a limited resource controller (instead of requiring the controller to run on a cloud server). Locally a wrapper (based on the concore library) shares data through the Mediator from PM and controller.

### F. USER EXPERIENCE

**FIGURE 11.**
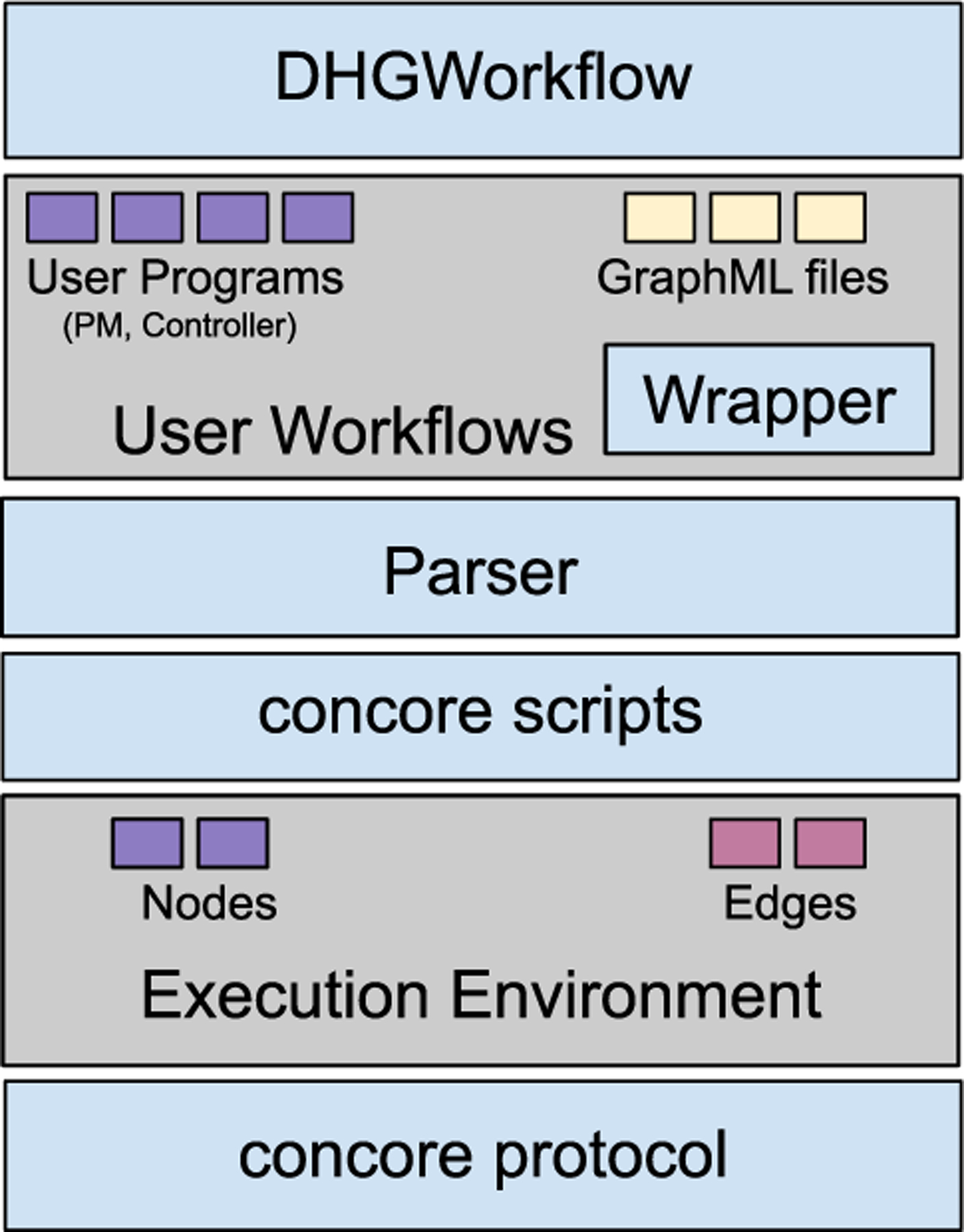
The CONTROL-CORE deployment architecture in the client sites.

Figure 11 shows the deployment architecture of the CONTROL-CORE framework, with its major components at the client sites. At its base is the concore protocol that specifies how the application developer develops the programs (such as PMs and controllers). It also consists of the concore scripts that enable the execution of the workflows, defining them as edges and nodes in the execution environment.

CONTROL-CORE consists of a browser-based open-source visual workflow composer, DHGWorkflow.^9^ DHGWorkflow functions as a user-facing front-end to create Directed Hypergraphs (DHGs), a superset of DGs, using its drag-and-drop interface. DHGWorkflow lets the users create workflows visually without having to manually write the workflow definitions (which is common in standard workflow languages such as CWL and WDL). DHGWorkflow stores the workflows as GraphML files in the file system storage, together with the concore user programs such as PMs and controllers. These GraphML files and the user programs collectively define the user workflows. CONTROL-CORE also consists of a parser to parse the GraphML files generated by DHGWorkflow into a python representation. Leveraging the Wrapper, CONTROL-CORE thus facilitates a seamless execution of the workflows locally in the execution environment or in a distributed manner.

As a GraphML implementation, DHGWorkflow is fully compatible with other GraphML frameworks, such as the popular downloadable GraphML tool, yEd [36]. yEd is a generic GraphML composer that allows users to design various types of GraphML diagrams. DHGWorkflow focuses entirely on composing hypergraph workflows, unlike yEd. It is simpler and easier to use by the CONTROL-CORE application developers. The DHG representation covers more potential application scenarios compared to DGs or DAGs.

DHGWorkflow comes with features for collaborations among the users, such as sharing a workflow with other collaborators through a URL or a workflow ID and collaboration on the same workflow by multiple users, by caching them in the server. It also tracks the history of changes made to the workflow by the users by storing the workflows created by the user in the cookies of the user’s browser. Therefore, any changes made to the workflow definition can be reverted. DHGWorkflow allows the workflows to be saved as GraphML files. DHGWorkflow also consists of other features such as i) a custom validation based on user-provided Javascript code snippets for nodes and edges, and ii) exporting the workflows as PNG and JPG images. The CONTROL-CORE Parser, developed using the python libraries lxml [37] and beautifulsoup4 [38], parses the GraphML files into their respective Python representations.

## IV. IMPLEMENTATION

This section looks into the implementation details of the CONTROL-CORE Mediator and wrappers and how they facilitate the distributed execution securely and seamlessly.

### A. MEDIATOR MULTITENANCY

The Mediator is a Python-based Docker container with a RESTful interface provided by Flask. Since Flask is not optimized to run stand-alone in production, we fronted it with Gunicorn, [39] a Python Web Server Gateway Interface. Gunicorn exploits the multiple processors and the multiple cores available in the server to cater to and process multiple REST calls at once. For example, a 16 processor 4-core server could run 64 worker nodes, efficiently parallelizing the workload. The Flask community has recommended such a deployment architecture rather than deploying a web application entirely based on Flask in a production environment. The website of the CONTROL-CORE project, delivered by a GET request, is hosted in the same deployment architecture of Flask and Gunicorn.

When deployed in a public cloud server, the Mediator can serve concurrent requests from multiple users. However, exposing the Mediator to the public as a cloud service creates a security concern, and consequently, a need for proper authentication measures. To manage the access to the Mediator better, we deploy Kong API Gateway [40] together with the Mediator. Kong is an open-source API gateway developed as an extension to NGINX [41] load balancer, with easily-configurable authentication, authorization, and access control measures. Each Mediator interface is exposed as a Kong API, composed of a service definition and a route definition. The service definition specifies where the backend service endpoint is. The route definition specifies how the users can access the respective service via the API exposed through Kong.

We limit access to the Mediator by firewall policies to only allow the ports exposed by Kong, hence providing access control measures. We also define the access to the admin APIs of Kong more restrictively. In the public cloud-based Mediator deployment, the firewalls are configured through the AWS security groups [42]. The Mediator exposes the service endpoints and the website at port 80 to the public.

As CONTROL-CORE aims to serve users who belong to a wide area network, representing several organizations, their data space must be protected and separated from others. We call these users “tenants” of the Mediator. Once the API keys are created from OSPARC by invoking the Kong’s admin interface, the tenants can build workflows using the programs such as PMs and controllers.

When the PMs are deployed in OSPARC, multiple tenants could access them simultaneously to create and execute workflows with controllers deployed across various sites. Similarly, tenants can compose workflows from different PMs and controllers deployed across multiple sites concurrently. The files belonging to each workflow are exchanged across the workflow’s services through a user-specified directory (we call it, dirname). The dirname must be included in all the service invocations to exchange the files in the Mediator correctly.

The APIs that communicate with the PM and controller wrappers should be secured to avoid compromising their access. We configure the key-auth plugin provided by Kong for those private APIs. Each user can create a unique Kong consumer and associated API key (which can be configured to expire after a specific time interval). Once a user creates an API key, it needs to be used in each service request. The API gateway will drop the REST calls without the correct API key. Hence, access requests to the Mediator private APIs without the API key will fail, hence protecting them from unauthorized access.

**FIGURE 12.**
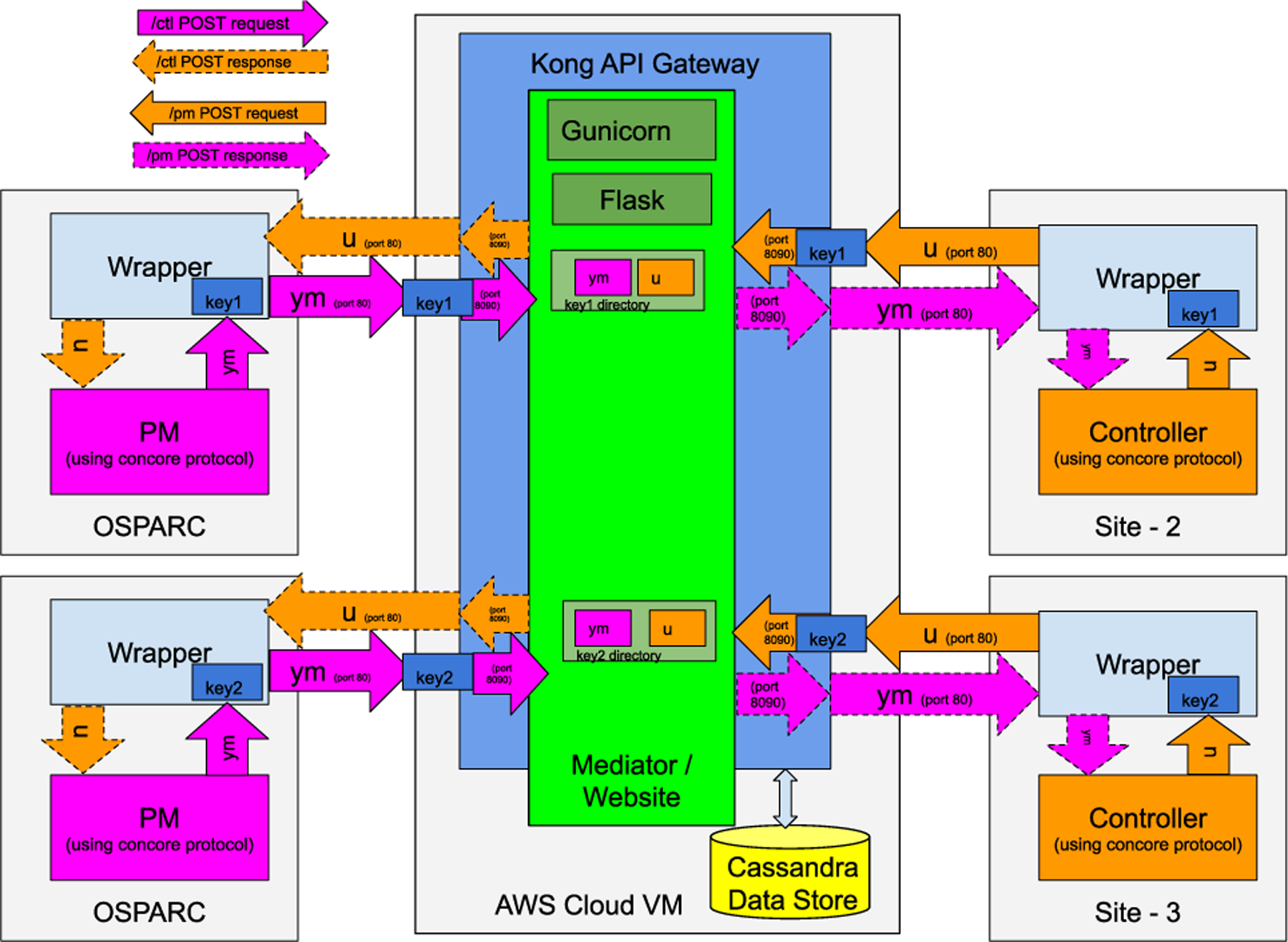
Multitenant deployment of CONTROL-CORE Mediator with the API Keys from Kong.

Figure 12 illustrates the multitenant execution of the Mediator with two tenants running their workflows concurrently via the Mediator. The Mediator is deployed with Kong in front, receiving all the requests first before forwarding them to the Mediator, as in a load balancer. Kong is configured with Apache Cassandra [43] as its data store. The data store persists the service and route definitions and the global configurations of the Kong API gateway, such as the timeout and plugin definitions. Site-2 and site-3 have the controllers communicating with the PMs in OSPARC via the Mediator in this representation. Several such sites may execute their workflows concurrently, between themselves and with those PMs hosted in OSPARC. Although OSPARC is presented here to host the PMs as in one typical use case scenario of the SPARC ecosystem, the PMs can be hosted on any site.

Relying on just the user-provided dirname alone to create a folder is unsuitable and unsafe for a multitenant environment. Multiple users may concurrently choose the same dirname for their workflows, as they are unaware of the existing directories that belong to the workflows of other tenants. Therefore, the Mediator stores the workflow’s data files (u and ym) in a directory dedicated to each workflow. The directory has a name with the user-provided dirname followed by the API key of the tenant, as below:

directory = dirname + “ _ “ + apikey


The Mediator includes an initialization method for the Mediator to acquire the startup values for the files u and ym. The security of the Mediator is limited access through the Kong API keys. AsCONTROL-CORE uses files to transfer data between the PM and controller through the Mediator, we also should consider the security of the host or the container that consists of the Mediator. The Mediator uses the secure_filename construct to ensure that a tenant cannot maliciously or naïvely replace the system files or any other file that does not belong to the tenant’s workflow through the RESTful POST invocations of the APIs. This approach prevents the files from being written to a folder in a higher level in the folder hierarchy or a folder outside from where the Mediator initializes, by disabling the characters such as “/” and “..” in the dirname and replacing them with safe characters such as “_” instead. The Mediator returns a success message in JSON [44] format when the method invocation completes successfully. The status output message from the Mediator is parsed and interpreted by the programs such as the PM and controller.

### B. DISTRIBUTED API DEFINITIONS

Now, we look into the Mediator services, init, cleanup, ctl, and pm, and how they work with the API key (key) for the secured access and invocation of the APIs. The init service initializes the Mediator before the execution of a workflow for the first time. Upon invocation, the cleanup service cleans up the Mediator and removes the workflow files stored in Mediator after the workflow completion. The ctl service endpoint of the Mediator lets the invocation of the controller program, whereas the pm service endpoint allows the invocation of the PM program.

The init and cleanup services share a similar API endpoint that do not produce a file output. However, the init service expects the initial values of the files passed on as the body of the HTTP POST request. ∀ service ∈ {init, cleanup}, the REST API endpoint is at /service/<dirname>?apikey=<key>. For example, a sample POST request to invoke the init service endpoint of dirname, “test” with an API key, “xyz”: /init/test?apikey=xyz.

The init procedure is currently invoked from PM, although it can be invoked from either PM or controller. This procedure provides the Mediator with the initial values of the files shared between the service nodes before the service APIs such as ctl and pm are invoked. The cleanup procedure periodically empties the folders after the workflow executions are completed. This procedure ensures that the temporary outputs from the service APIs such as ctl and pm are not left behind in the server of the Mediator. The cleanup procedure can be run from one of the clients as the final step.

The pm and ctl services also need to indicate which files they are expecting as the output/return file from the invocation. Therefore, ∀ service ∈ {pm, ctl}, the endpoint is at /service/<dirname>?fetch=returnfile& apikey=<key>.

For example, POST request to invoke the ctl service endpoint with dirname of “test,” using an API key, “xyz” is: /ctl/test?fetch=u&apikey=xyz. This indicates the file u must be fetched via the Mediator and returned. The respective pm service endpoint is, /pm/test?fetch=ym&apikey=xyz.

The wrappers use the concore protocol to connect to the local node, and Python requests module to connect to the remote node. After using concore to read the data locally, the local file that passed that data is then used to create a POST request to the proper URL (denoted as MEDIATOR in the cwrap.py code presented in the Appendix.). This request also includes the API key and service name (for example, the wrapper that connects to the controller will request the/pm service). Unlike concore.unchanged, the Mediator cannot ensure that the data has changed. There is a loop that repeatedly issues POST requests until the simulated time t has changed. If the POST requests time out, the loop reissues the POST requests and only fails after an agreed-upon global time delay. Typically, the loop exits and the wrapper does concore.write to continue the closed-loop.

The Mediator can occasionally encounter a race condition and deadlock when u (or ym) is accessed concurrently in the Mediator by the /ctl and /pm interfaces. The problem happens when read access is attempted by one POST response simultaneously to write access by the other POST request. The race condition is more frequent with Gunicorn due to multiple worker processes. Although the PM and its respective wrapper, as well as the controller and its respective wrapper, are synchronized by the concore module, the Mediator does not have the concore module based serialization. We use Python FileLock library for safe inter-process communication when multiple processes access the same files in the Mediator. This library provides a mutual exclusion (mutex) [45] to the file access to prevent concurrent read-write accesses, thus resolving these race conditions.

### C. SECURE ADMIN INTERFACE

Since each user needs to have their own API key to access the secured APIs of Mediator, there should be a straightforward approach for the generation of consumers and API keys. Typically, the Kong API Gateway’s admin interface is private and is not exposed to the public, as that would be a security concern. With public access to the admin interface, malicious users may take down the API gateway or naïve users may misconfigure the APIs and break the deployment. We limit access to API key generation through only the OSPARC platform by configuring the firewall policies in the security group of the CONTROL-CORE VM in the AWS. However, even then, we should not expose the whole admin interface of the Kong as-is to prevent curious users from accessing the entire configuration. Therefore, we expose the API key creation interface of Kong securely through a *secondary Kong* as a service, only to the OSPARC network.

**FIGURE 13.**
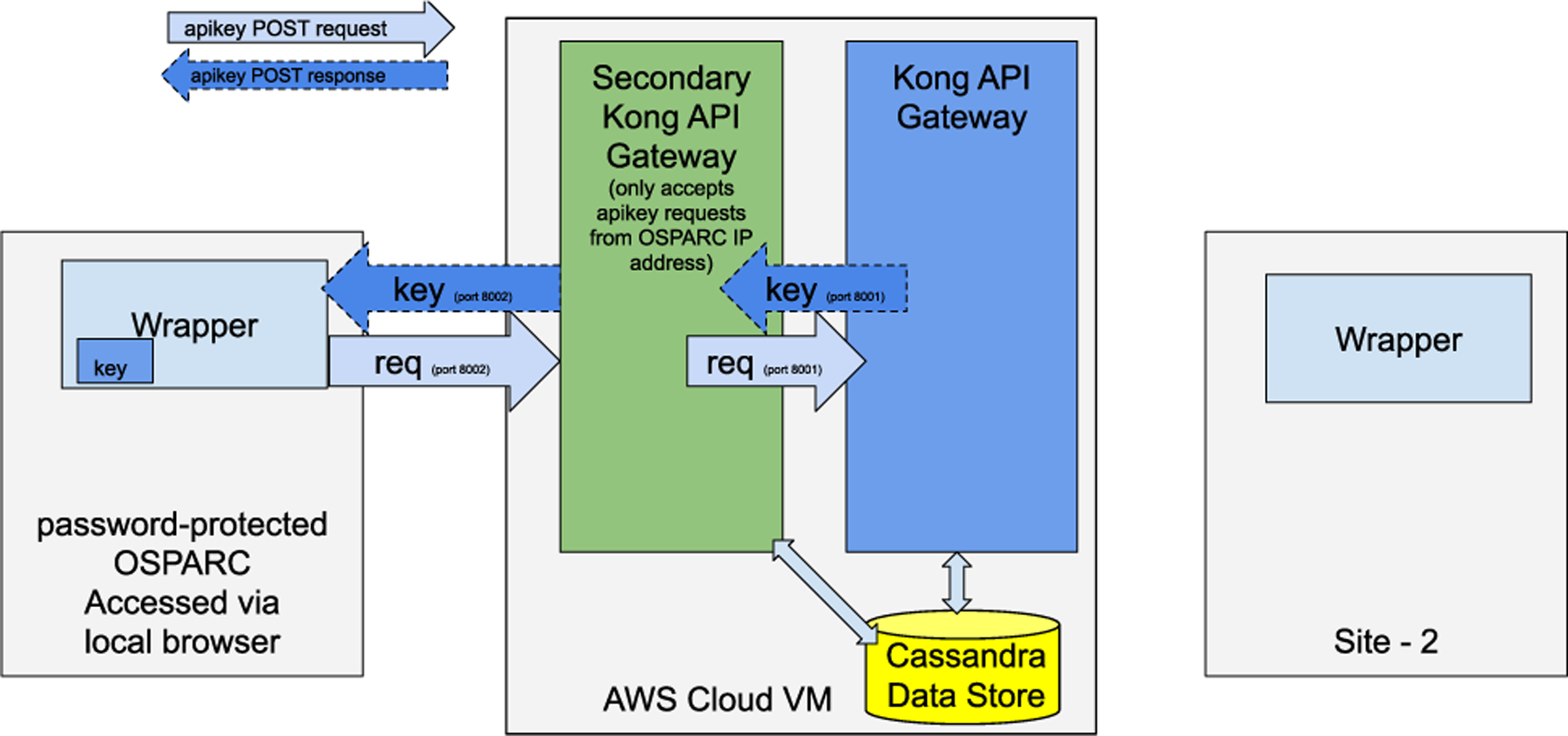
Kong’s admin interface securely exposed as a service to OSPARC network through another Kong.

Figure 13 shows the CONTROL-CORE deployment, with the Mediator configured with two Kong instances. Here the Kong is configured to generate API keys (port 8002) only when requested from the specified networks such as OSPARC. We highlight that this secondary Kong is utilized only when the API keys are generated. An *API key generator* feature in-built in the CONTROL-CORE PM wrappers creates a consumer and an API key for the user if the user does not have an API key.

Suppose the user already has a valid API key generated in the previous iteration. In that case, the API key generator will return the existing key to the user rather than creating a new one. Because users invoke the API key generator by logging into OSPARC through their local browsers, each user can copy and paste their unique key to their other wrapper instances (such as the controller wrapper in site-2 depicted in Figure 13) and the wrapper instances of different workflows. The admin interface for the API key generation does not require to be reaccessed by the user as long as the API key remains valid and the user possesses them in their wrappers. Hence, once the API key is generated and copied to the PM and controller nodes, the secondary Kong is not accessed by the same user in most cases for a long time. Therefore, the impact of the secondary Kong on the performance is negligible.

## V. EVALUATION

We evaluate the functionality and performance of CONTROL-CORE with a set of illustrative use cases for closed-loop neuromodulation control systems. We assess CONTROL-CORE across various programming languages and execution environments in a local and distributed execution.

### A. MPC WITH LINEAR PM

To get an idea of how a realistic neurostimulation simulation performs on various platforms using concore, we initially used a linear non-pulsatile model for a rat cardiac PM:

(1) x(t+1)=Ax(t)+Bu(t)ym(t+1)=Cx(t)+Du(t)

The model is defined by four configuration matrices: **A**, **B**, **C**, and **D**. The column vector **x** is the internal state of the PM. The column vector **u** has six elements (three stimulations points: Vagal, Sympathetic, and Baroreceptive; each stimulation point is defined by a frequency and pulse width). The output of the PM is a column vector with two elements, Heart Rate (HR) and Mean Arterial blood Pressure (MAP). The simulation time, *t*, is an integer indicating the number of heartbeats since the start of the simulation corresponding to concore.simtime. This is a non-pulsatile model since it only reports HR and MAP every heartbeat, rather than giving detailed information on the varying pressure during the systole and diastole periods. In contrast, the complete pulsatile model [46] requires solving Delayed Differential Equations (DDEs) [47], which are challenging to do efficiently in Python or Octave.

Even though (1) is highly simplified compared to the complete pulsatile model, (1) is usable with the controller [46] designed using Model Predictive Control (MPC) [48]. MPC can handle multi-input and multi-output systems, which are usually hard to be handled by scalar single-loop feedback controllers [49]. It also can handle constraints. Constraints are crucial for the biomedical system because violating them can lead to unsafe consequences. MPC has preview capability over an extended period. MPC uses a flexible and open methodology for solving optimization problems, making it extendable in many ways. MPC consists of certain challenges in the development of proper models: Sometimes, a complex nonlinear model and a disturbance/noise model are required, which is especially significant for biomedical systems. Other limitations include a selection of prediction horizon and weight matrices, and the design of extra conditions to guarantee stability should be carefully considered. Choosing MPC for the controller here is only an example—concore is compatible with other approaches.

This controller can be implemented in Python using cvxopt [50], requiring about 0.07 seconds per heartbeat on a 2.6GHz laptop. For benchmarking purposes (1) has the advantage that it can easily be implemented in Python with concore (notes: initialization is omitted; X contains matrices obtained from a data file set up using pmcvxpymatcore.dir; conversions to/from Numpy arrays and transpositions .T are required after reading and before writing so that Plant can work with column vectors whilst concore needs lists):


**import** concore 
**import** numpy as np
# initialize X and internal state (x) omitted
**def** Plant(u, x, X):
    newx = np.dot(X['A'], x) + np.dot(X['B'], u)
    newy = np.dot(X['C'], x) + np.dot(X['D'], u) 
    **return** newx, newy 
concore.delay = 0.02
init_simtime_u = "[0.0,0.0,0.0,0.0,0.0,0.0,0.0]" 
init_simtime_ym = "[0.0,0.0,0.0]"
ym = np.array([concore.initval(init_simtime_ym)]).T 
**while**(concore.simtime<concore.maxtime): 
    **while** concore.unchanged() :
         u = concore.read(1, "u", init_simtime_u) 
    u = np.array([u]).T 
    x, ym = Plant(u, x, X) 
    concore.write(1, "ym", **list**(ym.T[0]), 1)


We benchmarked this with five platforms: OSPARC (hosted in the AWS cloud infrastructure with northern Virginia cloud region), an x86 (2.6 GHz, 8 GB, Windows 10) laptop (in eastern Pennsylvania), an x86 (2.8 GHz, 16 GB, macOS Big Sur) laptop (in northwestern Georgia), a virtual machine (3.6 GHz, 8 GB, Windows 10) with Matlab license (hosted in eastern Pennsylvania) and AWS cloud (hosted in northern Virginia). We initially tested the cloud deployment with t2.micro AWS VM because it is free-tier eligible (1 GB, 1 vCPU, Linux). However, we observed that AWS t2.micro was of too poor performance, and instead, we upgraded to an r5.large instance (2 vCPU, 10 ECU, 16 GB, Linux). This AWS platform is used for (local) Docker computation and Mediator communication. There are two classes of benchmarks: local (that do not use the Mediator) and distributed (that use the Mediator for communication but have computation performed elsewhere).

Table 2 shows the local benchmarks for the linear cardiac model (1) using the cvxopt controller. The speed of the local implementation depends on several factors, including concore.delay and the time (independent of concore) for numerical computations performed by the particular controller/PM on the given hardware. Inside of concore.read is concore.retrycount that indicates the number of times reads had to be reattempted due to zero-length files. Although concore is functional for an arbitrarily short concore.delay, we use the heuristic that concore.retrycount should be around 10 percent of the number of simulation cycles. On most local platforms, this gives a concore.delay around 0.005–0.02 seconds, which represents the minimum time overhead incurred by each edge in the GraphML file.

**FIGURE 14.**
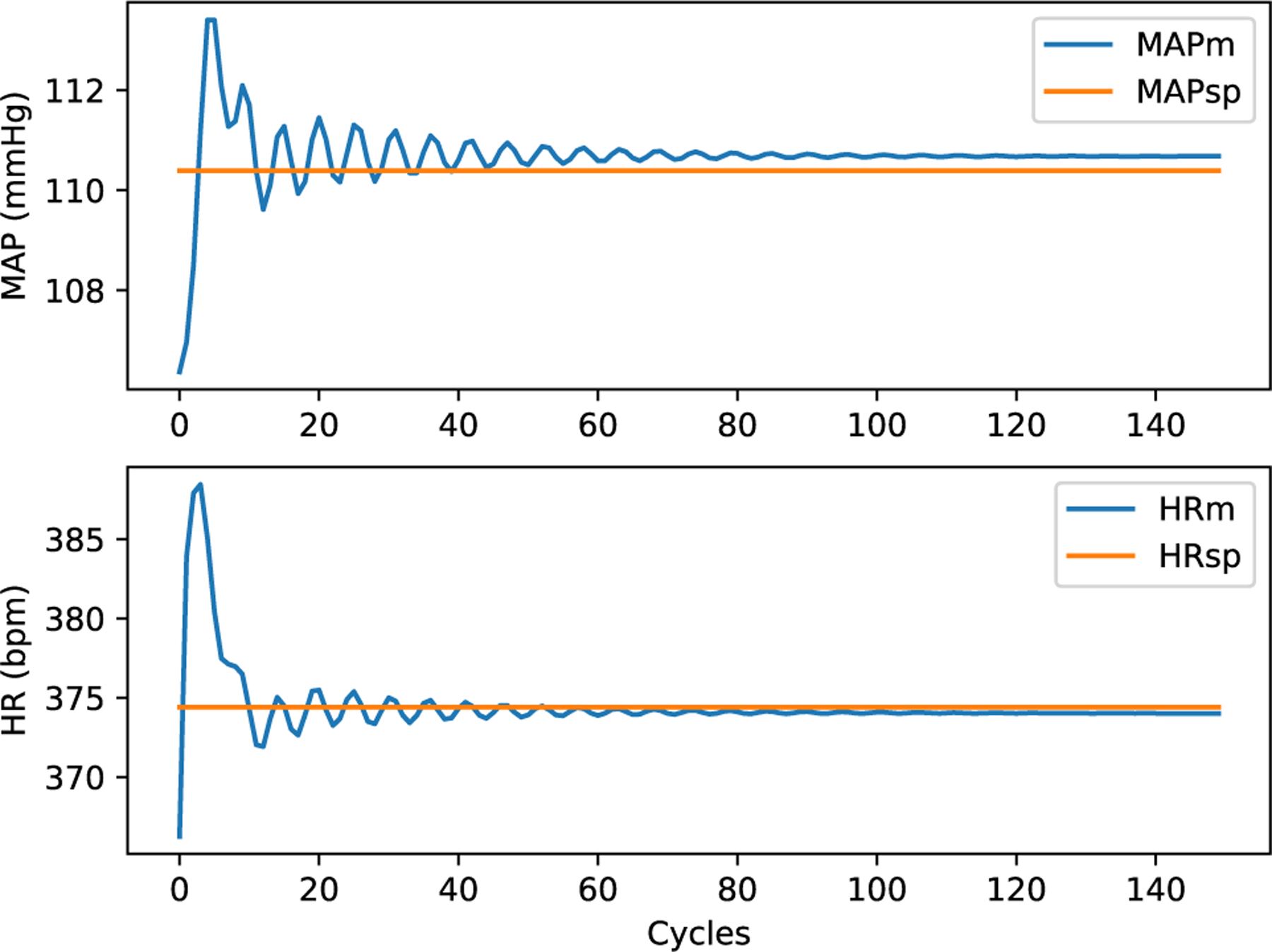
HR and MAP (ym) of Linear Model.

**FIGURE 15.**
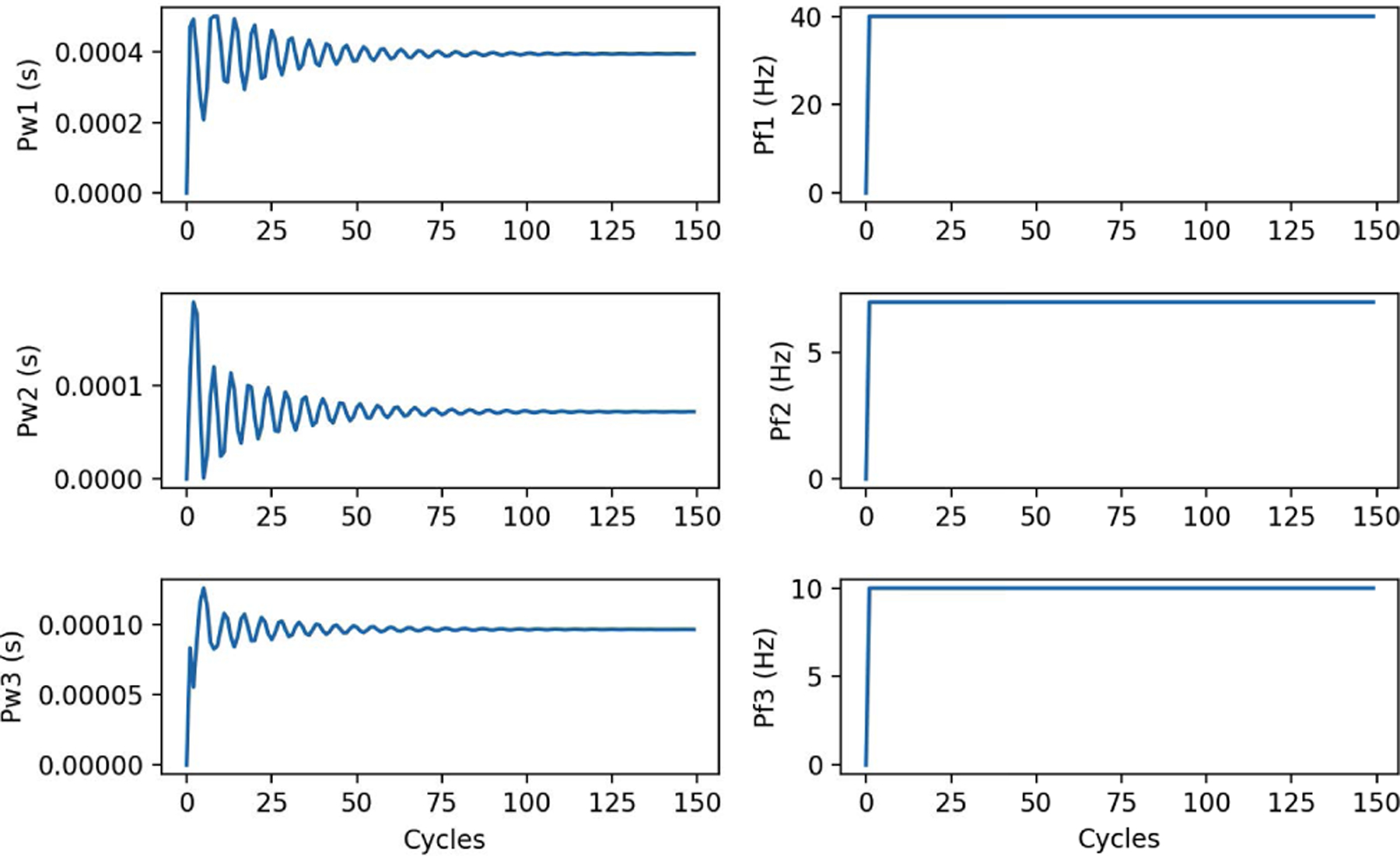
Stimulation output (u) of Linear Model.

On OSPARC, there are two possible local implementations: a) using the built-in sidecar file sharing implicit with nodes and edges created in the OSPARC UI (like Figure 3); b) a makestudy implementation using a GraphML file created outside of OSPARC whose nodes and edges are invisible to the OSPARC UI. For all the benchmarks, the controller gives plots of HR and MAP (Figure 14 obtained from ym) and stimulations (Figure 15 obtained from u). Table 3 shows the benchmarks for the corresponding distributed executions.

**FIGURE 16.**
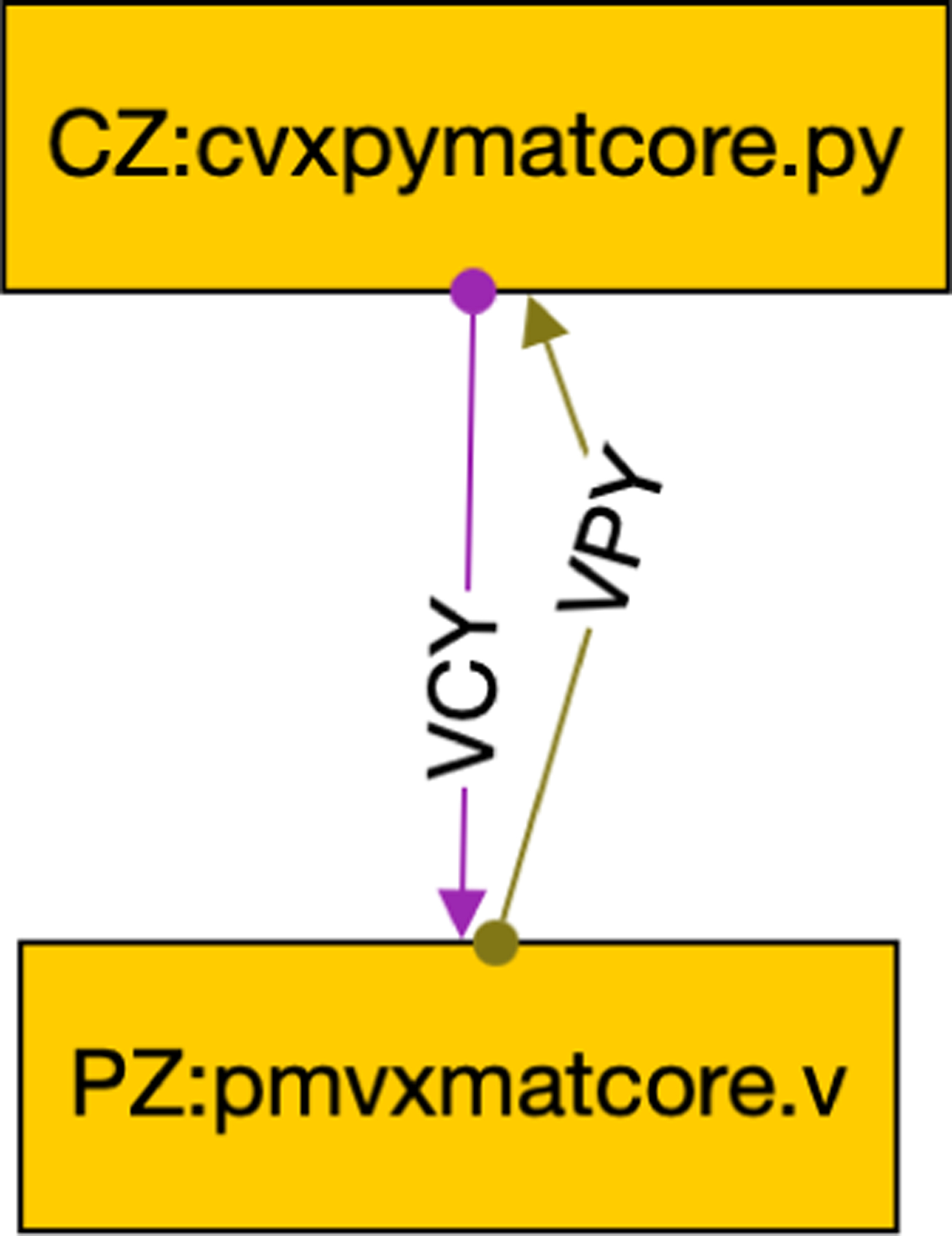
Python controller with Verilog PM.

**FIGURE 17.**
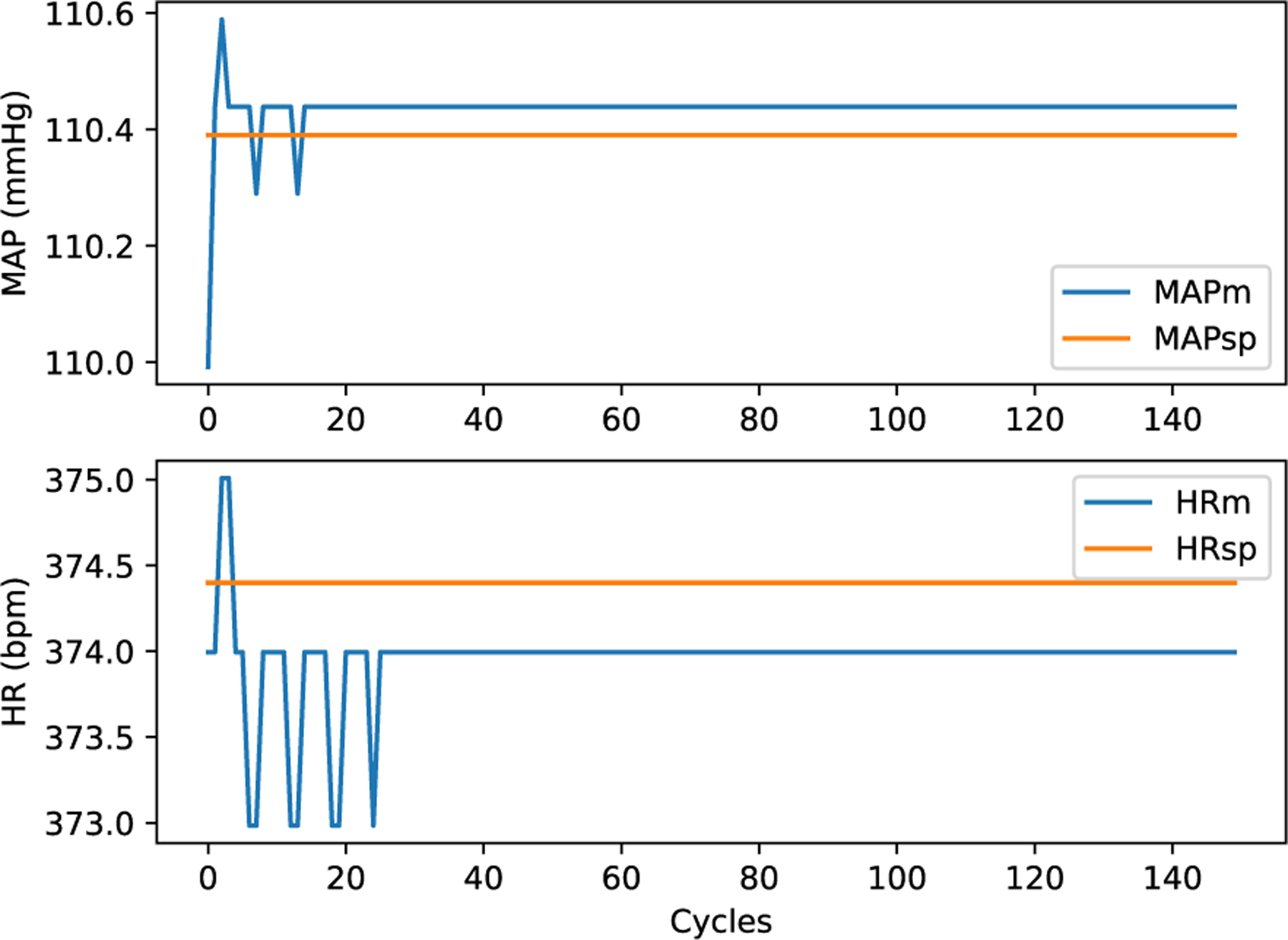
HR and MAP (ym) of Linear 16-bit Verilog.

One of the advantages of CONTROL-CORE is the ability to connect nodes written in different languages. Figure 16 shows the same Python controller connected to a PM using a 16-bit matrix coprocessor [51] coded in Verilog to realize (1). The HR and MAP in Figure 17, which is more zoomed into the region near the setpoint (110.4 mmHg, 374.5 bpm) than the other figures, exhibits quantization because the 16-bit format, which has a 9-bit mantissa, cannot reflect small perturbations to numbers of this magnitude. CONTROL-CORE allows users to explore such tradeoffs.

### B. MPC WITH PULSATILE PM

**FIGURE 18.**
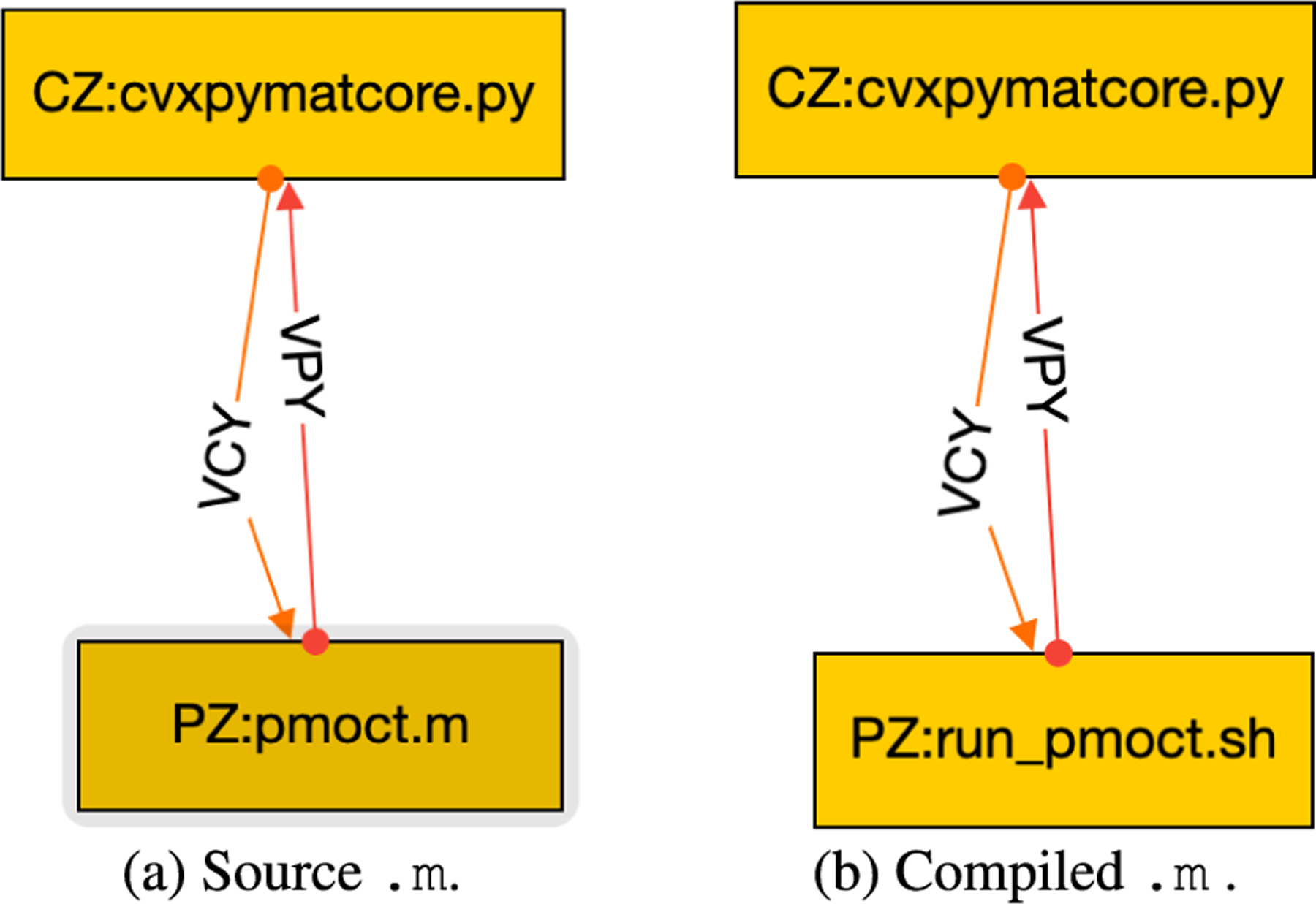
Pulsatile Matlab to solve DDEs.

**FIGURE 19.**
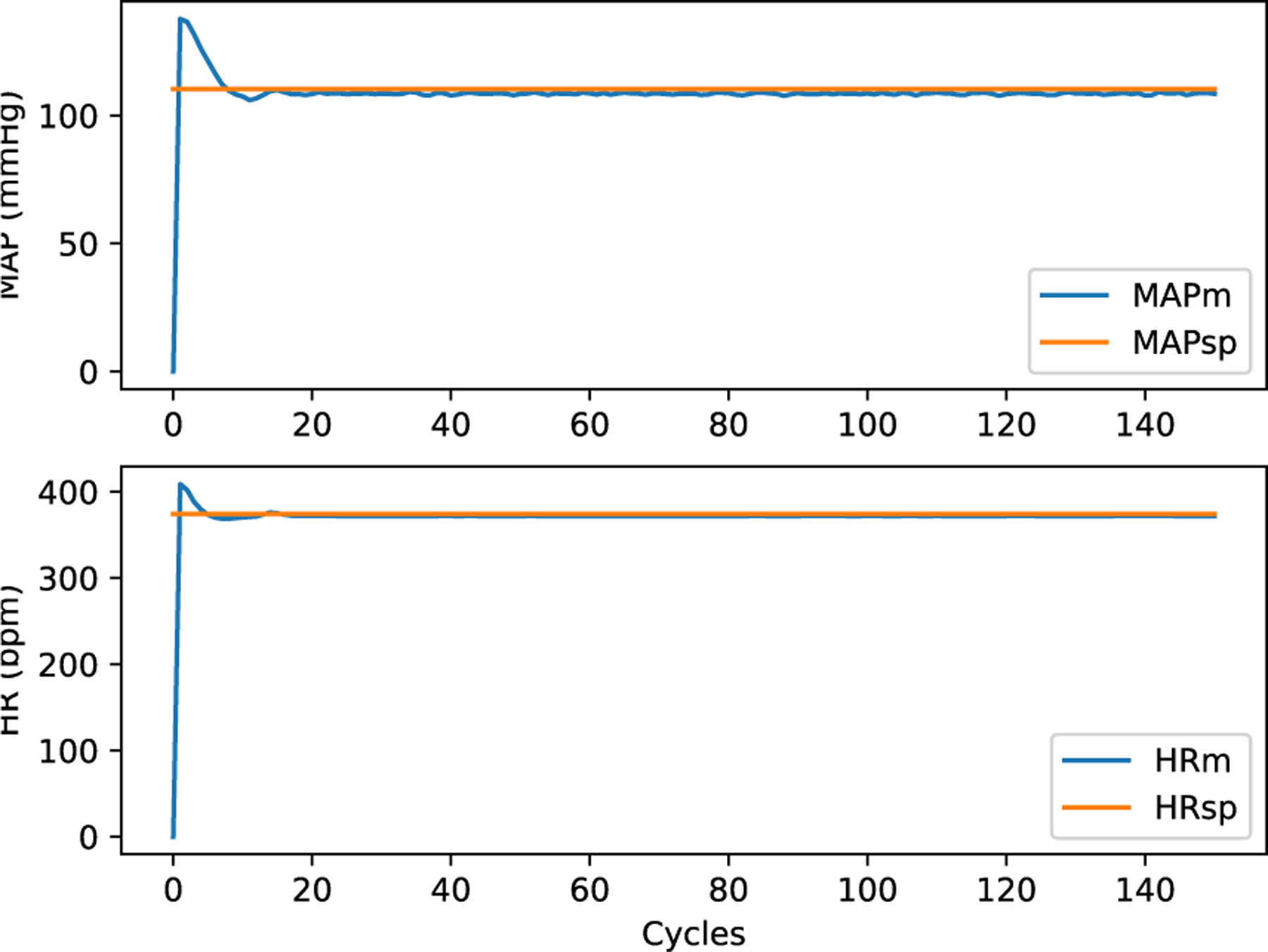
HR and MAP (ym) of Pulsatile Model.

**FIGURE 20.**
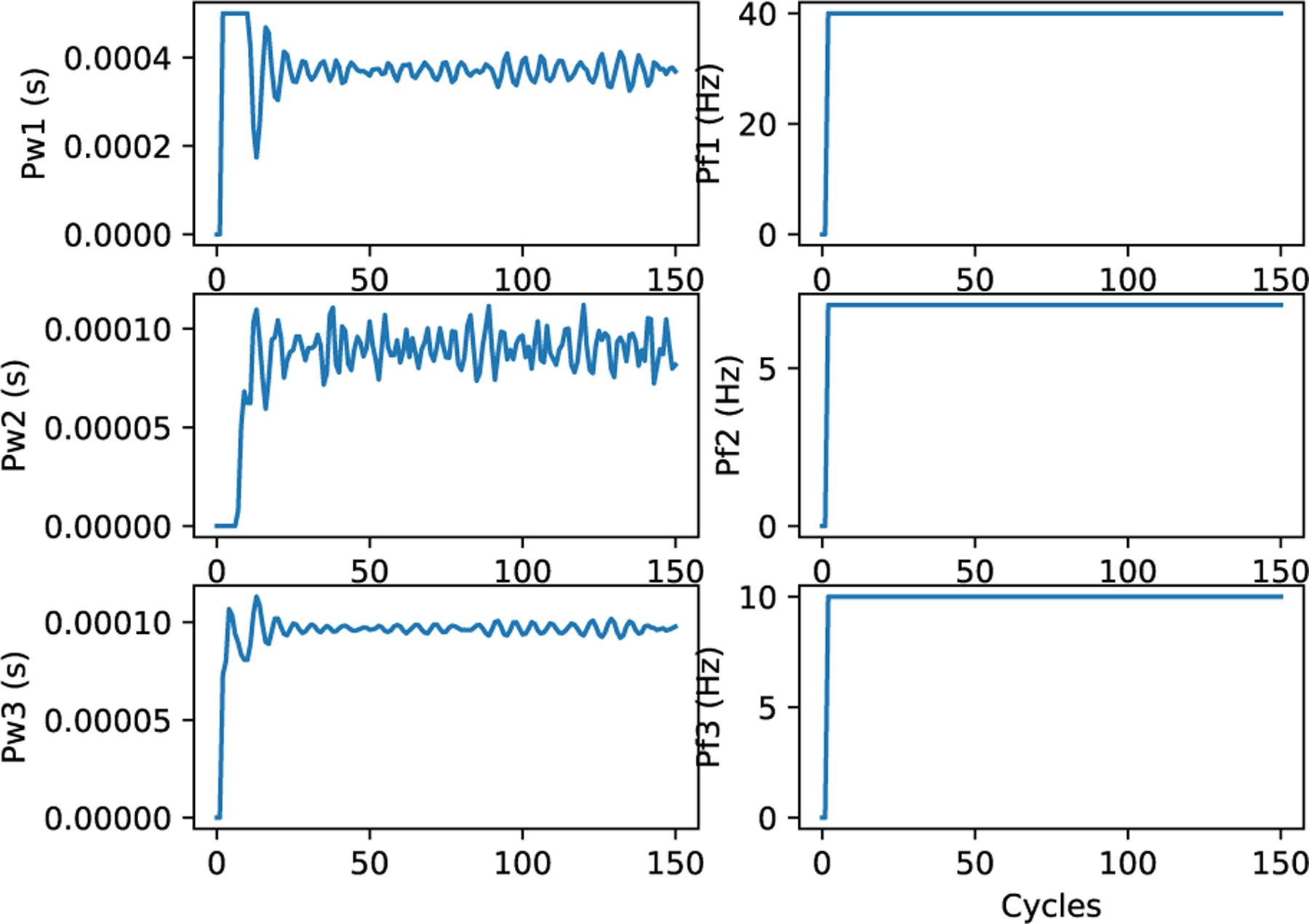
Stimulation output (u) of Pulsatile Model.

Using (1), which is the basis for pmcvxpymatcore.py and pmvxpymatcore.v as depicted in Figures 4 and 16, does not yield a realistic simulation. Instead, the full pulsatile model [46] gives a more accurate simulation, as shown in Figure 18 using pmoct.m written in proprietary Matlab to solve DDEs. Figure 18a, which interprets the source code file, is only possible on local systems that possess a Matlab license. To overcome this restriction, the local Ubuntu version provides a ./compile command that invokes MCR. The resulting object code and its invoking script, run_pmoct.sh, can be used by local Ubuntu systems without the Matlab license as shown in Figure 18b. This can then be containerized and used on any platform (without a Matlab license) using Docker. Although setting this up requires a license and a little effort on the developer’s part, once compiled, it can run on any local operating system that supports Docker without a Matlab license. The simulation output in Figures 19 and 20 is noticeably different than the earlier examples because the fidelity of the PM is better. The simulation speed (about two iterations per second) is slower because of the time required to solve the DDEs.

### C. LSTM WITH PULSATILE PM

**FIGURE 21.**
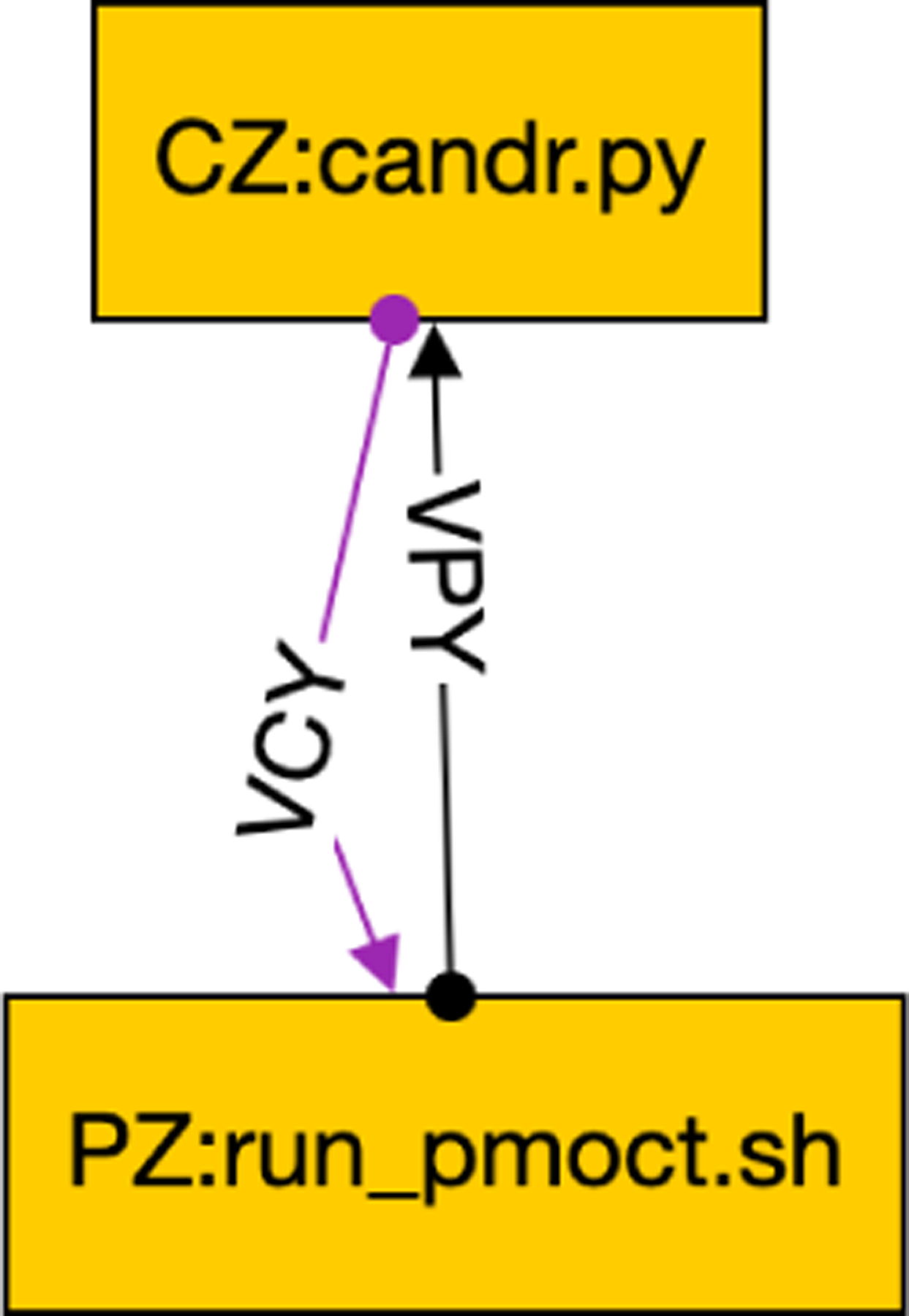
LSTM controller with Pulsatile PM.

Other controller formulations are possible, for example, a Tensorflow [52] controller (candr.py) based on LSTM neural network trained from experimental data (or in this case from random exploration of the Pulsatile PM), as shown by Figure 21. Tensorflow-LSTM controller is an MPC wherein the model is an LSTM. In addition to the advantages of the MPC, this consists of an added benefit of learning from data as opposed to developing equations and fitting parameters, depending on the problem. Once trained, the model structure and the weights are stored in an .h5 file (inside candr.dir). For Docker, the source-file directory needs to include a special candr.Dockerfile since Tensorflow is not included in the default.^10^

**FIGURE 22.**
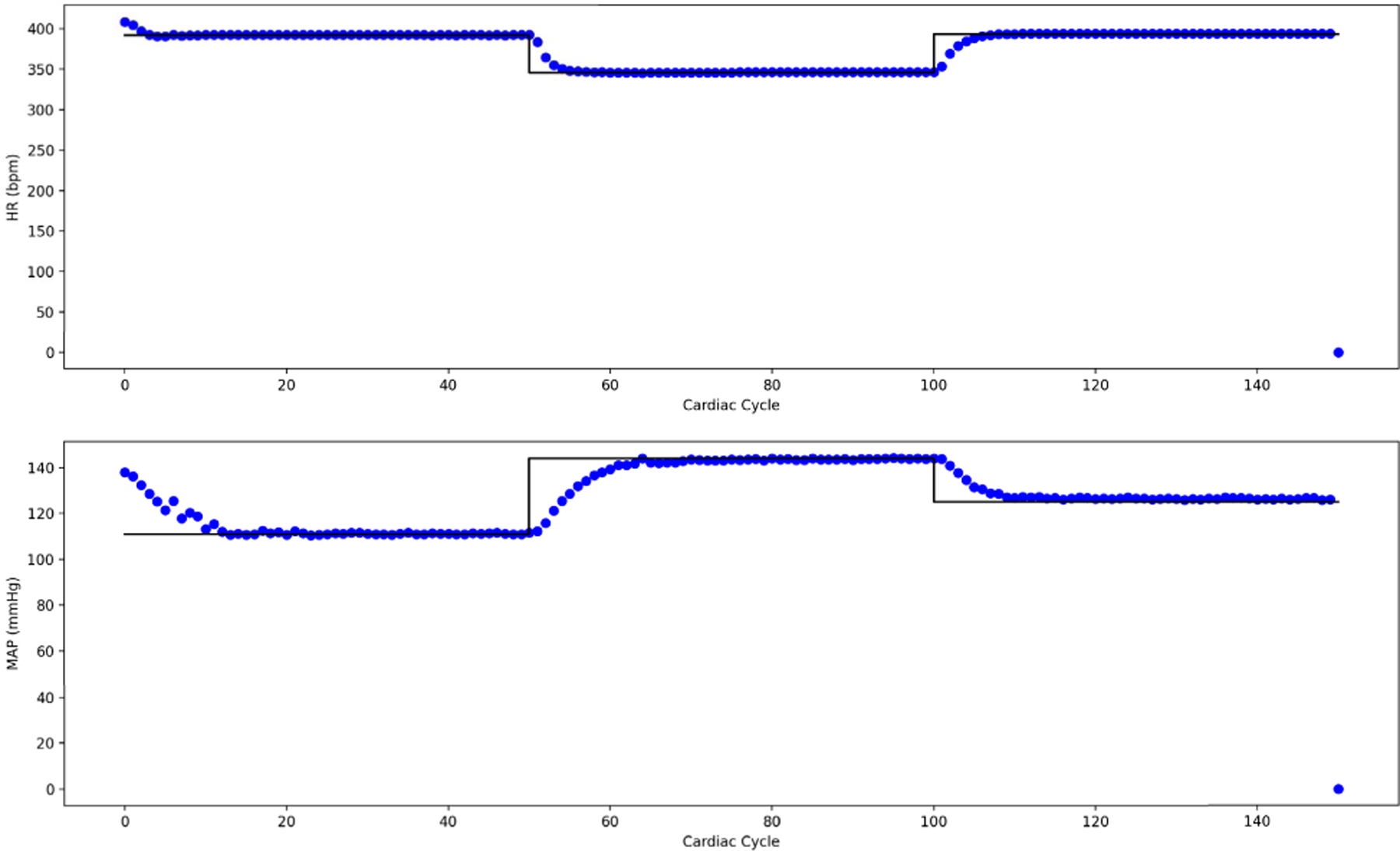
HR and MAP (ym) of LSTM controller.

**FIGURE 23.**
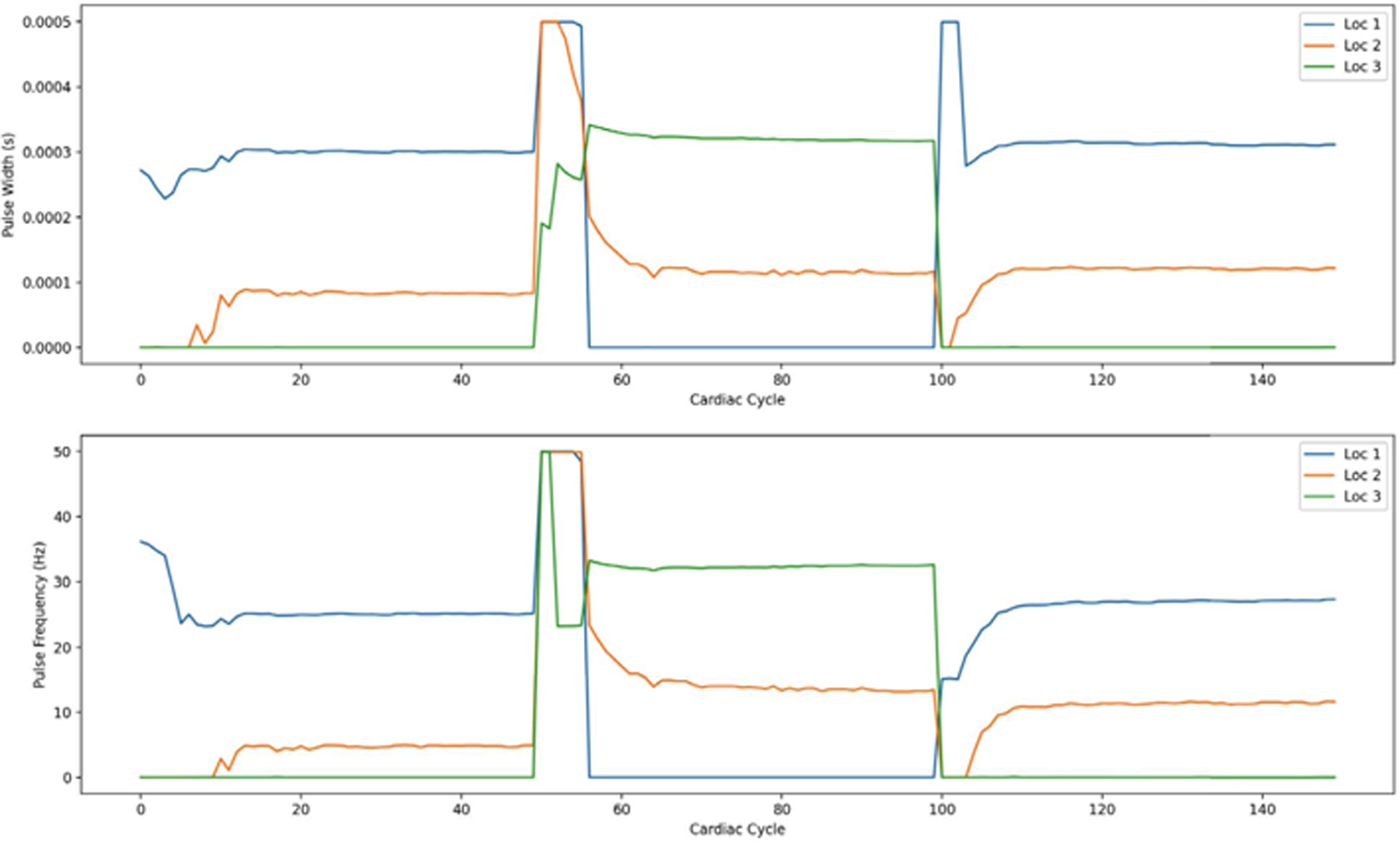
Stimulation (u) of LSTM controller.

Figure 22 shows the HR and MAP generated by CONTROL-CORE for three different set points, and Figure 23 gives the corresponding stimulation parameters. The speed is about half of MPC with the Pulsatile PM.

### D. POWER-AWARE TOOLS

**FIGURE 24.**
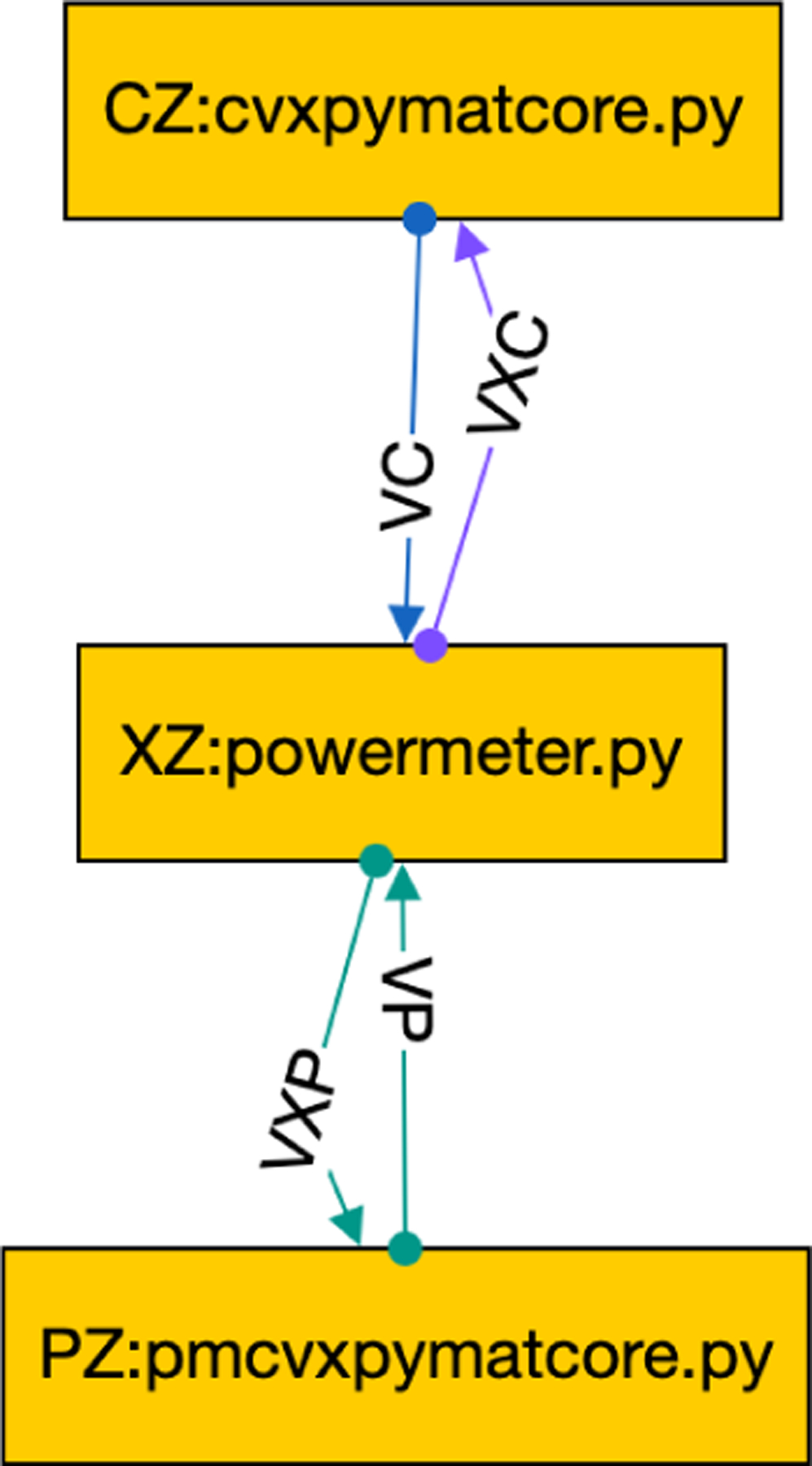
Power Meter connects Controller and PM.

One of the goals of the CONTROL-CORE platform is to allow control system designers to explore the tradeoffs of different alternatives. A metric of interest for implanted controllers is the power they consume, which can be attributed to several factors, including the actual neural stimulation, measurement, communication, and computing. For example, Figure 24 illustrates a powermeter.py tool that connects between the example controller and PM previously illustrated in Figure 4. This node passes the input (u) from the VC edge unchanged to the VXP edge while also computing the energy that would be required to accomplish this. For full communication, it also passes VP back to VXC while noting the HR. (This example ignores the energy required to measure MAP and HR, although similar code could consider measurement and communication costs.)


**while**(concore.simtime<concore.maxtime): 
    **while** concore.Unchanged():
        u = concore.read(concore.iport['VC'], "u", init_simtime_u) 
    concore.write(concore.oport['VXP'], "u", u) 
    **while** concore.unchanged() :
        ym = concore.read(concore.iport['VP'], "ym", init_simtime_ym) 
    concore.write(concore.oport['VXC'], "ym", ym) 
    **if** ym[1] != 0:
       period = 60/ym[1] 
       energy += period *
         (u[0]*u[1]+u[2]*u[3]+u[4]*u[5])


Because the power meter node (XZ) has two input ports and two output ports, we disambiguate which edge is connected to which port using concore.iport and concore.oport. For example, the first concore.read takes its input from VC and the second one takes its input from VP. It does not matter what port numbers are assigned to these edges. Similarly, the output edges VXC and VXP are referenced symbolically.

The period of each beat can be computed from HR (ym[1]), and the energy of each of the three stimulation points is the product of its pulse width and frequency times that period. The advantage of this approach is neither the controller nor PM needs to be instrumented for conducting such power-aware simulations. Indeed, they could just as easily have been pre-built Docker images. Users simply edit the GraphML file to invoke a simple tool like this. Users could also easily create similar tools for their own customized experiments in their language of choice using pre-built PM and/or controller images without knowing about the internal details of the images, which might have been written in a language that is unavailable or unfamiliar to the users.

### E. DISCUSSION

Stability is a critical factor in control systems [53], especially in the applications that aim to support medical use cases such as CONTROL-CORE. concore operates in a time-sensitive manner, providing synchronization between the PMs and Controllers. An event-triggered control scheme is an approach to address communication and computation constraints of real-time control tasks that are designed to be implemented on embedded processors. The goal of event-triggered control schemes, as opposed to time-triggered control strategies, is to increase the functionalities of the embedded processors using real-time scheduling algorithms based on event-triggered execution of control tasks [54].

Efficient real-time scheduling of event-triggered control tasks is proposed in the literature, such as the *H*_∞_ weighted integral event-triggered synchronization [55]. An1example of a sample event-triggered scheduling algorithm is to execute a control task whenever a specific error gets larger than a threshold or the state norm. Instead of periodic implementations, this aperiodic (event-triggered) implementation of control tasks saves the microprocessors’ computation and communication resources and increases their durability and functionalities. Using concore protocol in designing event-triggered control strategies is feasible. Implementing and deploying more control systems with CONTROL-CORE, including the event-triggered control systems, is future work.

## VI. CONCLUSION

We have designed a scalable multitenant simulation and design platform for closed-loop peripheral neuromodulation control systems. Through several illustrative use cases, we demonstrated the capabilities of the CONTROL-CORE platform to model closed-loop neuromodulation control systems to treat conditions affecting internal organs. As part of the larger SPARC program, CONTROL-CORE fills a gap in the potential to leverage PMs for developing closed-loop VNS systems by allowing interaction between control algorithms and the PMs. With the novel concore protocol that facilitates seamless communication between the controllers and the PMs and a secured Mediator deployment, the CONTROL-CORE platform provides a modular architecture for composing workflows from various disjoint services locally or remotely. Our evaluations on the use case prototypes highlight the feasibility and performance of the proposed framework.

Standard workflow frameworks are not optimized to run across multiple infrastructures in a distributed manner. The CONTROL-CORE Mediator facilitates a distributed workflow execution based on RESTful communication across the service nodes. While the Mediator is still a centralized entity, we note that such a RESTful approach can be extended to support completely decentralized workflows across multiple servers and infrastructures. With a centralized (such as the API key generation of CONTROL-CORE) or a decentralized trust mechanism, closed-loop workflows can be adopted into a decentralized peer-to-peer environment as future work. Hence, we believe CONTROL-CORE provides the first step towards decentralized modeling of peripheral neuromodulation control systems globally across diverse infrastructures through RESTful interactions.

## Figures and Tables

**TABLE 1.** SPARC Ecosystems.

Component	Functionality
K-CORE	Ontology of medical terminology.
DAT-CORE	Experimental data from animal and human experiments.
MAP-CORE	Mapping of geometric, neural, and systemic relationships of organs.
SIM-CORE	Computerized simulations of PMs (O^2^S^2^PARC).

**TABLE 2.** Local Linear Cardiac Benchmark.

Platform	OS	PM	file sharing	time/iteration
Laptop	macOS/Win	.py	symbolic link	0.05 – 0.02
Laptop	macOS/Win	.m	symbolic link	0.08
Virtual	Win	.py	symbolic link	0.06
Virtual	Win(Matlab)	.m	symbolic link	0.09
AWS	Docker	.py	volume	0.05 – 0.03
AWS	Docker	.m	volume	0.06
OSPARC	JupyterLab	.py	sidecar	2.0 – 3.0
OSPARC	JupyterLab	.py	symbolic link	0.03

**TABLE 3.** Distributed Linear Cardiac Benchmark.

Controller Platform	file sharing	PM Platform	file sharing	time/iteration
Laptop (PA)	link	Laptop (GA)	link	0.25
Laptop (PA)	link	OSPARC (VA)	sidecar	5.0

## Footnotes

1
https://itis.swiss/

2 Emory University School of Medicine, Lehigh University, San José State University, the University of Pittsburgh, and Thomas Jefferson University.

3
https://github.com/ControlCore-Project/concore

4 C++ uses the vector class. Since Verilog does not have an equivalent, its compatible but more cumbersome calling sequence is omitted here.

5 Note that the unlabeled edges in Figure 3 denote the sidecars that allow the transmission of the “u” and “ym” files of Figure 1b. The edges do not represent the files themselves. The edges could allow additional files to be transmitted in a more complex example.

6 Like Figure 3, the edges VCY and VPY do not represent files “u” and “ym” of Figure 1b, but rather the means by which they are transmitted.

7 The non-Docker implementation, which does not have this restriction, follows an analogous approach for consistency.

8
https://github.com/ControlCore-Project/Mediator

9
https://github.com/controlcore-project/DHGWorkflow

10 candr.py requires particular versions of Tensorflow and Numpy, which illustrates the advantage of containers for reproducible results [3] compared to a local installation that might have incompatible versions.

## APPENDIX

### A. BUILD SCRIPTS

The Posix ./build script with Figure 4 as its GraphML:

mkdir CZ
cp ./src/cvxpymatcore.py ./CZ/cvxpymatcore.py
cp ./src/concore.py ./CZ/concore.py
cp ./src/cvxpymatcore.iport ./CZ/concore.iport
cp ./src/cvxpymatcore.oport ./CZ/concore.oport
cp -r ./src/cvxpymatcore.dir/* ./CZ
mkdir PZ
cp ./src/pmcvxpymatcore.py ./PZ/pmcvxpymatcore.py 
cp ./src/concore.py ./PZ/concore.py 
cp ./src/pmcvxpymatcore.iport ./PZ/concore.iport 
cp ./src/pmcvxpymatcore.oport ./PZ/concore.oport 
cp -r ./src/pmcvxpymatcore.dir/* ./PZ 
mkdir VCY 
mkdir VPY 
**cd** CZ
ln -s ../VCY out1 
**cd** .. 
**cd** PZ
ln -s ../VPY out1 
**cd** .. 
**cd** cz
ln -s ../VPY in1 
**cd** .. 
**cd** PZ
ln -s ../VCY in1 
**cd** ..

Here is the corresponding ./build script generated by makedocker:

mkdir docker-cvxpymatcore 
**cd** docker-cvxpymatcore
cp ../src/Dockerfile.cvxpymatcore Dockerfile 
cp ../src/cvxpymatcore.py . 
cp ../src/concore.py .
cp ../src/cvxpymatcore.iport concore.iport 
cp ../src/cvxpymatcore.oport concore.oport 
cp -r ../src/cvxpymatcore.dir/* . 
sudo docker build -t docker-cvxpymatcore . 
**cd** ..
mkdir docker-pmcvxpymatcore 
**cd** docker-pmcvxpymatcore
cp ../src/Dockerfile.pmcvxpymatcore Dockerfile 
cp ../src/pmcvxpymatcore.py . 
cp ../src/concore.py .
cp ../src/pmcvxpymatcore.iport concore.iport 
cp ../src/pmcvxpymatcore.oport concore.oport 
cp -r ../src/pmcvxpymatcore.dir/* . 
sudo docker build -t docker-pmcvxpymatcore . 
**cd** ..

### B. WRAPPER IMPLEMENTATION

The following illustrates the main aspects for cwrap.py:

**while** (concore.simtime<concore.maxtime) : 
     **while** concore.unchanged() :
          u = concore.read(1, name1, init_simtime_u) 
     f = {'file1' : **open**(concore.inpath+ '1/'+namel, 'rb') }
     r = requests.post(MEDIATOR + '/pm/'+sharedsecret+apikey+'&fetch='+name2, files=f, timeout=timeout_max)
     t=literal_eval(r.text)[0] 
     timeout_count = 0 
     t1 = time.perf_counter() 
     **while** oldt==t **or len** (r.content) ==0: 
          time.sleep(concore.delay)
          f = {'file1' : **open** (concore. inpath+ ' 1/’+namel, 'rb **'**)}
          r = requests.post(MEDIATOR + '/pm/ '+sharedsecret+ apikey+'&fetch='+name2, files=f, timeout = timeout_max) 
          timeout_count += 1
        **if** r.status_code**!=200 or** time.perf_counter()-t1 > 1.1*timeout_max: 
             **print**("timeout or bad POST request "+str(r.status_code)) 
             quit()
        t=literal_eval(r.text)[0] 
oldt = t 
oldym = r.text 
concore.write(1, name2, oldym)
